# A generalized stoichiometric model of C_3_, C_2_, C_2_+C_4_, and C_4_ photosynthetic metabolism

**DOI:** 10.1093/jxb/erw303

**Published:** 2016-08-17

**Authors:** Chandra Bellasio

**Affiliations:** Department of Animal and Plant Sciences, University of Sheffield, Sheffield, UK

**Keywords:** Assimilation, bioengineering, carbon-concentrating mechanism, constraint, dark reactions, flux balance, flux-balance analysis, NAD-ME, NADP-ME, PEPCK

## Abstract

The goal of suppressing photorespiration in crops to maximize assimilation and yield is stimulating considerable interest among researchers looking to bioengineer carbon-concentrating mechanisms into C_3_ plants. However, detailed quantification of the biochemical activities in the bundle sheath is lacking. This work presents a general stoichiometric model for C_3_, C_2_, C_2_+C_4_, and C_4_ assimilation (SMA) in which energetics, metabolite traffic, and the different decarboxylating enzymes (NAD-dependent malic enzyme, NADP-dependent malic enzyme, or phosphoenolpyruvate carboxykinase) are explicitly included. The SMA can be used to refine experimental data analysis or formulate hypothetical scenarios, and is coded in a freely available Microsoft Excel workbook. The theoretical underpinnings and general model behaviour are analysed with a range of simulations, including (i) an analysis of C_3_, C_2_, C_2_+C_4_, and C_4_ in operational conditions; (ii) manipulating photorespiration in a C_3_ plant; (iii) progressively upregulating a C_2_ shuttle in C_3_ photosynthesis; (iv) progressively upregulating a C_4_ cycle in C_2_ photosynthesis; and (v) manipulating processes that are hypothesized to respond to transient environmental inputs. Results quantify the functional trade-offs, such as the electron transport needed to meet ATP/NADPH demand, as well as metabolite traffic, inherent to different subtypes. The SMA refines our understanding of the stoichiometry of photosynthesis, which is of paramount importance for basic and applied research.

## Teaser

A model based solely on mass-balance constraints refines our understanding of the trade-offs, energy requirements, leaf-level fluxes, and plasticity mechanisms in different biochemical types of assimilation.

## Introduction

Interest in engineering a biochemical carbon-concentrating mechanism (CCM, abbreviations listed in Table 1) to suppress photorespiration in crops is increasing (Furbank *et al.*, 2015; Long *et al.*, 2015). The metabolic activities of a CCM are shared between mesophyll (M) and bundle sheath (BS) cells. Structurally, the leaf parenchyma is organized in concentric layers of cells, with an outer mesophyll encircling one or two layers of BS cells. In some species, the BS cells are isolated from the surroundings by a gas-tight suberized cell wall (Lundgren *et al.*, 2014). Biochemically, the compartmentalization of glycine decarboxylase (GDC) activity in the BS allows plants to take advantage of photorespiratory CO_2_ release (Sage *et al.*, 2012; Mallmann *et al.*, 2014), giving rise to a mechanism that delivers CO_2_ around the Rubisco in the BS – the so-called C_2_ shuttle (Schulze *et al.*, 2013; Keerberg *et al.*, 2014). The ‘C_4_ cycle’ is a further adaptation involving an energy-dependent carboxylation‒decarboxylation cycle. CO_2_ is initially ﬁxed into four-carbon (C_4_) organic (amino) acids in the M by phosphoenolpyruvate (PEP) carboxylase (PEPC). These then diffuse to the BS where they are decarboxylated. C_4_ plants have traditionally been grouped into three subtypes (Table 2) according to the main decarboxylating enzyme, NAD-dependent malic enzyme (NAD-ME), NADP-dependent malic enzyme (NADP-ME), or phosphoenolpyruvate carboxykinase (PEPCK) (Hatch, 1987), but there is considerable diversity in the degree of engagement of the biochemical activities of the CCM between subtypes. For instance, maize (*Zea mays*) has been shown to operate two BS decarboxylation enzymes (NADP-ME and PEPCK) and two CO_2_ delivery pathways (via malate, MAL, or aspartate, ASP, respectively) (Furumoto *et al.*, 1999, 2000; Wingler *et al.*, 1999). There is also overlap between BS and M functions. In maize, sucrose is synthesized mainly in the M, while starch is generally accumulated in the BS, although both possess enzymes to synthesize starch (Rascio *et al.*, 1980; Spilatro and Preiss, 1987). Both the BS and M reduce 3-phosphoglyceric acid (PGA). Further, pyruvate phosphate dikinase (PPDK) has traditionally thought to be confined to the M; however, PPDK was shown to be present and active in the BS (Aoyagi and Nakamoto, 1985; Majeran *et al.*, 2010), although the role of PPDK in the BS remains elusive.

**Table 1.** Acronyms, definitions, and variables. Quantities with dimensions are consistent with assimilation (μmol m^−2^ s^−1^).

*A*	Net assimilation
ALA, *ALA*	Alanine, ALA diffusion rate
ASP, *ASP*	Aspartate, ASP diffusion rate
ATP*, ATP*_TOT_*, ATP*_BS_*, ATP*_M_	Adenosine triphosphate, rate of ATP demand: total, in the BS, or in the M respectively
BS	Bundle sheath
CCM	Carbon-concentrating mechanism
CEF	Cyclic electron flow
CS*, CS*_TOT_*, CS*_BS_*, CS*_M_	Carbohydrate synthesis, rate of carbohydrate synthesis: total, in the BS, or in the M, respectively
DHAP, *DHAP*	Dihydroxyacetone phosphate, DHAP diffusion rate, respectively
*DHAP*_RPP_, *DHAP*_RPPM_, *DHAP*_RPPBS_	Rate of DHAP entering the RPP, in the M, or in the BS respectively
*F*	Rate of photorespiratory CO_2_ release $F=0.5\;V_{\text{O}}$
*f*_C_, *f*_O_, *f*_RLIGHT_, *f*_PR_, *f*_CS_, *f*_PPDK_	Input parameters defining, relative to total, the BS fraction of: Rubisco rate of carboxylation, Rubisco rate of oxygenation, Respiration in the light, PGA reduction, carbohydrate synthesis, and PPDK activity, respectively
*GA*	Gross assimilation (*A* + *R*_LIGHT_)
GDC*, GDC*_TOT_, *GDC*_BS_, *GDC*_M_	Glycine decarboxylase, GDC reaction rate: total, in the BS, or in the M, respectively
GLA	Glycolic acid
GLY, *GLY*	Glycine, GLY diffusion rate, respectively
*L*	Leak rate, i.e. magnitude of CO_2_ flux diffusing out of the BS, Eqn S19
LEF	Linear electron flow (flow of electrons derived from the photo-oxidation of water)
M	Mesophyll
MAL, *MAL*	Malate, MAL diffusion rate, respectively
MDH*, MDH*_BS_ , *MDH*_M_	Malate dehydrogenase, MDH reaction rate in the BS or M, respectively
ME*, ME*	Malic enzyme, ME reaction rate, respectively
*NADPH*_TOT_ *, NADPH*_BS_	NADPH demand: total or in the BS, respectively
OAA, *OAA*	Oxaloacetate, OAA diffusion rate, respectively
PCO	Photosynthetic carbon oxygenation (cycle), also known as photorespiratory cycle
PEP	Phosphoenolpyruvate
PEP, *PEP*	Phosphoenolpyruvate, PEP diffusion rate, respectively
PEPC	Phosphoenolpyruvate carboxylase
PEPCK*, PEPCK*	Phosphoenolpyruvate carboxykinase, PEPCK reaction rate, respectively
PGA, *PGA*	3-phosphoglyceric acid, PGA diffusion rate, respectively
PGLA	2-phosphoglycolic acid
PPDK*, PPDK*	Pyruvate phosphate dikinase, PPDK reaction rate, respectively
PR*, PR*_TOT_*, PR*_BS_*, PR*_M_	PGA reduction, reduction rate: total, in the BS, or in the M, respectively
PYR, *PYR*	Pyruvate, PYR diffusion rate, respectively
*R*	Rate of CO_2_ and NH_3_ release in the BS associated with the operation of the C_2_ shuttle, Eqn S16
*R*_LIGHT_ *, R*_LIGHT BS_ *, R*_LIGHT M_	Respiration in the light: total, in the BS, or in the M, respectively
*r*_O/C_	Input defining leaf-level Rubisco rate of oxygenation relative to carboxylation, also referred to as φ or *V*_O_/*V*_C_
*r*_PEPCK_	input parameter defining the activity of PECK relative to *V*_P_
RPP	Reductive pentose phosphate (cycle); also known as Calvin‒Benson‒Bassham cycle or photosynthetic carbon reduction cycle
Rubisco	RuBP carboxylase oxygenase
RuBP	Ribulose–1,5–bisphosphate
RuP	Ribulose–5–phosphate
*RuP*_phosp_, *RuP*_phosp M_, *RuP*_phosp BS_	Rate of RuP phosphorylation: total, in the M, or in the BS, respectively
SER, *SER*	Serine, SER diffusion rate, respectively
SMA	Stoichiometric model of assimilation
T*, T*	Transamination, Transamination rate
*V*_C_, *V*_CM_, *V*_CBS_	Rubisco rate of carboxylation: total, in the M, or in the BS, respectively
*V*_O_, *V*_OM_, *V*_OBS_	Rubisco rate of oxygenation: total, in the M, or in the BS, respectively
*V*_P_	PEPC rate of carboxylation
αKG	alpha–Ketoglutarate

**Table 2.** Functional classification of photosynthetic types and example species.

Type	C_3_	Proto-Kranz	C_2_	C_2_+C_4_			C_4_				
Subtype	-	-	-	NADP-ME	NAD-ME	PCK	NADP-ME	NADP-ME (+PCK)	NAD-ME	PEPCK (NADP-ME)	PEPCK (NAD-ME)
Example	*Triticum aestivum*	*Heliotropium procumbens*	*Mollugo verticillata*	*Flaveria pubescens*	*Alternanthera tenella*	*Alloteropsis semialata*	*Sorghum bicolor*	*Zea mays*	*Panicum* sp. (sensu stricto)	*Alloteropsis semialata* ssp. *semialata*	*Spartina* sp.
GDC compartmentalization	no	partial	full	full	full	full	full	full	full	full	full
Rubisco compartmentalization	no	partial	partial	partial	partial	partial	full	full	full	full	full
PEPC engagement	no	no	no	partial	partial	partial	full	full	full	full	full
PEPCK engagement	no	no	no	no	no	partial	no	partial	no	full	full
MDH engagement in the M	no	no	no	partial	no	partial	full	full	no	potentially active	no

Detailed quantification of ATP and NADPH supply and demand in the BS and M is critical for understanding the physiology and regulation of photosynthesis. In terms of supply, the partitioning of ATP and NADPH production varies between cell types depending on the light available locally in the BS or M (Bellasio and Griffiths, 2014*c*). The dependence of ATP generation upon anatomical traits in the evolutionary continuum from C_3_ to C_4_ has recently been studied (Bellasio and Lundgren, 2016), and will not be addressed here. In this work, I shall concentrate on ATP and NADPH demand.

Quantifying ATP and NADPH demand in the M and BS requires detailed mechanistic understanding of assimilatory biochemistry. Mathematical modelling offers valid support for integrating knowledge at the systems level (Morandini, 2013; Singh *et al.*, 2014). Classical photosynthetic models have allowed the simulation of leaf-level assimilation in C_3_, C_2_, C_2_+C_4_, and C_4_ plants using a mechanistic description based on Rubisco PEPC kinetics (von Caemmerer, 1989, 2000, 2013). These models are based on several simplifications, which limit their applicability. First, they do not account for spatial segregation of biochemical processes, offering only limited support when the separate requirements of the BS and M are being studied. Second, classical model(s) do not distinguish between biochemical subtypes, making it difficult to evaluate the particular requirements of each subtype. Finally, the models were primarily developed to predict leaf-level CO_2_ exchange, while the stoichiometry, energetics, and fluxes between the BS and M, which represent a critical bottleneck for C_4_ photosynthesis (Pick *et al.*, 2011), are not treated sufficiently, and a dedicated model is consequently needed.

The aim of this work was to develop a stoichiometric model of assimilation (hereafter SMA) which (i) generalizes all pathways of assimilation (C_3_, C_2_, C_2_+C_4_, and C_4_ including C_4_ photosynthetic subtypes); (ii) is based only on stoichiometry and therefore does not rely on kinetic measurements; and (iii) includes all main photosynthetic reactions, but is user-friendly for non-specialists. The theoretical underpinnings of the SMA are described in detail and a range of simulations to exemplify the model rationale and behaviour are provided. Numerous topics are covered, including (i) an analysis of C_3_ and C_2_ photosynthesis and all subtypes of C_2_+C_4_ and C_4_ (NADP-ME, NAD-ME, and PEPCK, in different combinations) in operational conditions; (ii) the energetics involved in manipulating photorespiration in a C_3_ plant; (iii) the consequences of progressively upregulating a C_2_ shuttle in a background of C_3_ photosynthesis; and (iv) the consequences of progressively upregulating a C_4_ cycle in a background of C_2_ photosynthesis. Results quantify ATP and NADPH demand, which link dark and light reactions; refine our understanding of the stoichiometry of photosynthesis and the trade-offs inherent to different photosynthetic subtypes; and represent a useful framework for the integration of existing biochemical models.

## SMA development

**Fig. 1.**
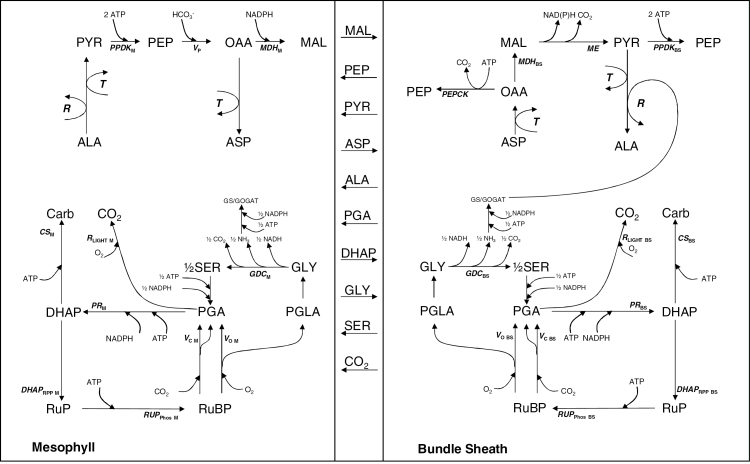
SMA schematic. The C_4_ CCM appears at the top, while C_3_ metabolism is at the bottom, partitioned between M and BS contributions. Metabolites for which fluxes are calculated are listed at the M–BS interface. The Excel workbook provided in Supplementary file 2 renders outputs according to this scheme.

The SMA was developed on the basis of a stoichiometric model of NADP-ME C_4_ photosynthesis (Bellasio and Griffiths, 2014*c*; McQualter *et al.*, 2016) to augment all the pathways of carbon assimilation in a single tool. The SMA calculates key reaction rates, and ATP and NADPH requirements, in the M and BS as well as fluxes between the BS and M when the following parameters are known: the locality of GDC and Rubisco, leaf-level Rubisco rates of carboxylation and oxygenation (*V*_O_, *V*_C_), and PEP carboxylation rate (*V*_P_). The ATP and NADPH requirements are SMA outputs and they are not related to light reactions at this stage. Reactions are typically grouped by the biochemical function of the pathways, of which only the entry point is calculated. The SMA is based only on well-established reaction stoichiometry. The SMA accounts for the interactions between C_2_ and C_4_ cycles, including the fluxes associated with amino group rebalancing, and for the NADPH and ATP demand of assimilatory processes. The theory underpinning the SMA can be followed in Fig. 1. Owing to space limitations, the full description of the SMA is reported in Supplementary file 1.

### Parameterization

The SMA has 12 input quantities (Table 3): three define Rubisco activity and assimilation (net assimilation, *A*; respiration in the light, *R*_LIGHT_; and Rubisco rate of oxygenation relative to carboxylation, *r*_O/C_), two define the activity of the CCM (PEPC engagement, as *V*_P_; and PEPCK activity relative to *V*_P_, *r*_PEPCK_), and seven partition key processes between the BS and M (*f*_C_, for Rubisco carboxylation rate; *f*_O_, for Rubisco oxygenation rate; *f*_GDC_, for glycine decarboxylase; *f*_RLIGHT_, for respiration in the light; *f*_PR_, for PGA reduction; *f*_CS_, for carbohydrate synthesis; *f*_PPDK_, for pyruvate phosphate dikinase). In addition, for NAD-ME subtypes it is possible to constrain malate dehydrogenase (MDH) activity to zero. Inputs can be constrained in different ways depending on the research questions. When the goal is to refine the analysis of a particular metabolic state of the leaf, input quantities may represent realistic biochemistry, otherwise inputs can be freely manipulated to simulate bioengineering or explore hypothetical scenarios. *A*, *R*_LIGHT_, and $r_{\text{O}/\text{C}}$ can be measured with accuracy (Bellasio *et al.*, 2014; Bellasio *et al.*, 2016*a*, *b*). Quantification of *V*_P_ is less straightforward, and can be achieved through an *in vitro* assay of PEPC activity (Pfeffer and Peisker, 1998). This is complicated by PEPC-sensitive regulation and feedback inhibition, and the consequent necessity of reproducing physiological metabolite and ion concentrations in the reaction mixture. In alternative *A*, $r_{\text{O}/\text{C}}$ and *V*_P_ can be predicted with biochemical models. Two types of formulations exist, either based on enzyme kinetics (and referred to as enzyme-limited), or based on the rate of ATP and NADPH made available by light reactions (and referred to as light-limited). Integrating enzyme-limited formulations in the SMA is straightforward, because the *A*, $r_{\text{O}/\text{C}}$, and *V*_P_ output by the biochemical model can be directly input into the SMA (see references below). Integrating light-limited formulations is more complicated and will be addressed in a dedicated paper. Physiological values are available for *r*_PEPCK_, gained through extensive biochemical work (Kanai and Edwards, 1999; Koteyeva *et al.*, 2015). The traits underpinning *f*_*O*_, *f*_*C*_, *f*_GDC_, and *f*_RLIGHT_ may require evolution or long acclimation periods to change (Sage *et al.*, 2012; Christin and Osborne, 2014) and are therefore considered constant during gas exchange experiments. In the SMA, *f*_*O*_, *f*_*C*_, and *f*_GDC_ represent the fraction of enzyme activity in the BS, rather than the physical distribution of the enzyme, but when enzyme compartmentalization is complete (Table 2), these become equal. The distribution of Rubisco and GDC can be quantified through proteomics, biochemical assays, or immunolocalization [e.g. Aoyagi and Nakamoto (1985), Majeran *et al.* (2005), and Keerberg *et al.* (2014)] and are generally known for model species (Edwards and Ku, 1987). Cases of intermediate GDC distribution are rare (Sage *et al.*, 2014), and, even in these cases, the enzyme distribution may be confidently taken as *f*_GDC_ because substrate concentrations and regulation may be similar in the M and BS. For Rubisco, when compartmentalization is incomplete (as in C_2_+C_4_ species), predicting *f*_*C*_ and *f*_*O*_ requires that the increased CO_2_ concentration in the BS be modelled, for instance using the validated models for C_4_, C_3_, C_2_, and C_2_+C_4_ photosynthetic subtypes (Farquhar *et al.*, 1980; von Caemmerer, 1989, 2000, 2013; von Caemmerer and Furbank, 1999). *f*_RLIGHT_ is often assumed to be 0.5 in C_4_ plants and 0.2 in C_2_+C_4_ plants (von Caemmerer, 1989, 2000), and may be determined from the relative BS/M mitochondrial abundance, or simply from the relative BS/M volume (Edwards and Ku, 1987). Circumstantial evidence suggests that *f*_CS_, *f*_PR_, *r*_PEPCK_, and *f*_PPDK_ may rapidly change and this has likely contributed to the difficulty of distinguishing them experimentally. Exact empirical parameterization may therefore be of limited interest, and it may be more informative to define physiological maxima and minima (Bellasio and Griffiths, 2014*c*). Within these limits, the adjustment of *f*_CS_, *f*_PR_, *r*_PEPCK_ and *f*_PPDK_ may help the plant maximize assimilation under transient environmental inputs, such as changes in light quality, which may unbalance the partitioning of ATP and NADPH supply (Bellasio and Griffiths, 2014*c*). The effect of varying these inputs will be simulated in the subsection below dedicated to C_4_ photosynthesis to show how these mechanisms can re-balance ATP and NADPH demand.

**Table 3.** Input quantities used in dynamic computer simulations shown in Supplementary Figure S1 and Figures 2–4

Simulation	1.2 C_3_ photorespiration	2.2 Proto-Kranz and C_2_	3.2 C_2_ + C_4_	4.2.1 Partitioning PGA reduction		4.2.2 Manipulating PEPCK activity		4.2.3 Partitioning carbohydrate synthesis		4.2.4 PPDK engagement in the BS	
Subtype	-	-	-	NADP-ME	NAD-ME	NADP-ME	NAD-ME	NADP-ME	NAD-ME	NADP-ME	NAD-ME
Figure	S1	2	3	4A	4B	4C	4D	4E	4F	4G	4H
**Basic quantities**											
*R*_LIGHT_ (μmol m^−2^ s^−1^)	1	1	1	1	1	1	1	1	1	1	1
*A* (μmol m^−2^ s^−1^)	9	9	9	9	9	9	9	9	9	9	9
*r*_O/C_	variable	0.45		0.05	0.05	0.05	0.05	0.05	0.05	0.05	0.05
**CCM**											
*V*_P_ (μmol m^−2^ s^−1^)	0	0	fitted for *L* = 0	10.85	10.85	10.85	10.85	10.85	10.85	10.85	10.85
*r*_PEPCK_	0	0		0	0	variable	variable	0	0	0	0
*MDH*_M_	irrelevant	Eqn S19	Eqn S19	Eqn S19	*MDH*_M_ = 0	Eqn S19	*MDH*_M_ = 0	Eqn S19	*MDH*_M_ = 0	Eqn S19	*MDH*_M_ = 0
**BS contribution**											
**Slow response**											
*f*_C_ *, f*_O_	0	fitted for *L* = 0	variable	1	1	1	1	1	1	1	1
*f*_GDC_	0	variable	1	1	1	1	1	1	1	1	1
*f*_RLIGHT_	0	0.2	0.5	0.5	0.5	0.5	0.5	0.5	0.5	0.5	0.5
**Fast response**											
*f*_PR_	0	0	0	variable	variable	0.25	0.25	0	0	0	0
*f*_CS_	0	0	0	0	0	0	0	variable	variable	0	0
*f*_PPDK_	0	0	0	0	0	0	0	0	0	variable	variable

## SMA simulations

The following simulations were selected to illustrate the capabilities, rationale, and behaviour of the SMA, while at the same time making some considerations of interest for the theme of this special issue. Simulations are grouped for photosynthetic types (C_3_, proto-Kranz and C_2_, C_2_+C_4_, and C_4_). For each type, two sets of simulations are presented: static scenarios, where the SMA is calculated for one set of inputs, representing realistic operational conditions (Supplementary Table S1), and dynamic simulations where inputs are varied (Table 3).

### Simulation 1. C_3_ photosynthesis

#### Simulation 1.1. Operational conditions

Outputs for C_3_ photosynthesis in operational conditions are shown in Supplementary Fig. S1. The C_4_ CCM is not operational. Carbon fixation and the reductive pentose phosphate (RPP) cycle operate in the M, while no carbon fixation is occurring in the BS. Although photorespiration and GDC activity are high, no glycine (GLY) is exported in the BS, and there is no net metabolite flux at the M–BS interface. All ATP and NADPH demand is in the M.

#### Simulation 1.2. Dynamic simulations

A dynamic scenario for a C_3_ photosynthetic type was simulated by varying *r*_O/C_ between 0 and 1 while keeping other quantities at 0 (Table 3). The resultant ATP and NADPH demand as a function of *r*_O/C_ is plotted in Supplementary Fig. S2A while the ratio between ATP and NADPH demand is plotted in Supplementary Fig. S2B.

### Simulation 2. C_2_ and proto-Kranz

#### Simulation 2.1. Operational conditions

Outputs for C_2_ photosynthesis are shown in Supplementary Fig. S3. To operate a C_2_ shuttle, GDC is absent in the M and the photorespiratory GLY produced in the M is decarboxylated in the BS and fixed by a small fraction of Rubisco located in the BS. Serine (SER) diffuses back to the M, exporting half of the GLY amino groups. The excess ammonia produced by GDC in the BS is fixed by glutamine synthetase/glutamine oxoglutarate aminotransferase (GS/GOGAT), transaminated to alanine (ALA), and diffuses back to the M so as to re-balance the amino groups. For the C_2_ shuttle to operate, an import flux of pyruvate (PYR) equimolar to *R* is required in the M. The C_4_ CCM is not operational. In this example, PGA is reduced mainly in the M and a substantial BS↔M triose phosphate exchange occurs. Because the CCM is not operational, there is no net export of NADPH from M to BS and the NADPH demand in BS of 1.1 μmol m^−2^ s^−1^ must be met by linear electron flow (LEF); however, cyclic electron flow (CEF) may be preponderant as the ratio of *ATP*_BS_ to *NADPH*_BS_ was ~8.

#### Simulation 2.2. Dynamic simulations

**Fig. 2.**
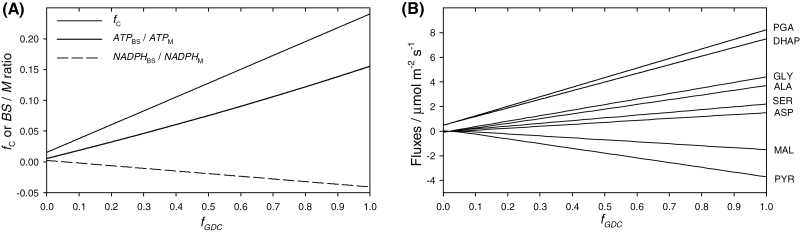
Simulation 2.2. From C_3_ to C_2_ photosynthesis. The BS partitioning of GDC activity (*f*_GDC_) was varied between 0 and 1. Panel (**A**) shows the partitioning of Rubisco activity that resulted in no net CO_2_ flux across the BS–M interface, together with the resultant ATP and NADPH partitioning. Panel (**B**) shows the corresponding metabolite fluxes. Inputs are shown in Table 3.

*f*_GDC_ was varied from 0 to 1 in an idealized C_3_ plant, thus simulating the transition to C_2_ photosynthesis through intermediate states of GDC compartmentalization, which are generally referred to as proto-Kranz (Sage *et al.*, 2014). At each *f*_GDC_ level, *f*_C_ was fitted so that CO_2_ leakage was zero (Supplementary Equation S34). The resultant *f*_C_ as a function of *f*_GDC_ is plotted in Fig. 2A. Values of *f*_C_ above the curve will result in a net influx of CO_2_ into the BS driven by Rubisco fixation, and in a CO_2_ concentration in the BS lower than that in the M. Values of *f*_C_ below the curve will result in a net CO_2_ efflux out of the BS, and in a CO_2_-concentrating effect of the C_2_ shuttle. Moving Rubisco and the photosynthetic carbon oxygenation (PCO) cycle to the BS results in changing the locality of ATP and NADPH demand (Fig. 2A) and requires a substantial traffic of metabolites (Fig. 2B). The flux of PGA out of the BS and the opposite flux of dihydroxyacetone phosphate (DHAP; which is lower than that of PGA by a value equal to carbohydrate synthesis, CS) results from setting *f*_PR_ = 0 in this simulation. This constraint also determines an excess of reducing power in the BS because the NADH produced by GDC in the BS is not used by PR. In these conditions the SMA predicts MAL to diffuse from the BS to M to shuttle the excess reducing power in the BS. MAL is oxidized to oxaloacetate (OAA) in the M and transaminated to ASP, which diffuses back to the BS and is transaminated back to OAA to supply MDH in the BS. The pair PYR/ALA balances the amino groups resulting from OAA/ASP transamination, and the amino groups resulting from the flux of GLY and SER are directly dependent on the operation of the C_2_ shuttle. Alternative scenarios may involve NADH resulting from GDC activity sustaining a minimal level of PR in the BS (shown in Supplementary Fig. S3).

### Simulation 3. C_2_+C_4_

C_2_+C_4_ photosynthesis has complete GDC compartmentalization to the BS and intermediate states of CCM engagement (Table 2), corresponding to moderate *V*_P_ and incomplete Rubisco compartmentalization. The CCM subtype is defined by the engagement of MDH in the M and PEPCK in the BS.

#### Simulation 3.1. Operational conditions

An example of NADP-ME C_2_+C_4_ is shown in Supplementary Fig. S4A. The C_4_ CCM is operational and CCM activity is sufficient to exceed Rubisco carbon fixation in the BS, and there is a net CO_2_ efflux from BS (*L* > 0). PR is mainly located in the M, and triose phosphate trafficking is higher than for the C_2_ photosynthetic type. Because a large part of Rubisco is mainly located with GDC in the BS, and photorespiration is reduced by the activity of the CCM, the effectiveness of the C_2_ shuttle (as *R*) is reduced relative to C_2_ photosynthesis. PR in the BS consumes all NADPH available through *MDH*_M_, therefore *T* ≈ 0; however, there is no residual NADPH deficit, and LEF is not required in the BS.

In a NAD-ME C_2_+C_4_ photosynthetic subtype (Supplementary Fig. S4B), the C_4_ CCM results in the same export of CO_2_ to the BS as in the NADP-ME C_2_+C_4_ photosynthetic subtype; however, MDH activity in the M is zero, all OAA is transaminated, and the CCM does not export reducing power to the BS. PR and glycolate recycling in the BS consumes NADPH at a rate of 2.32 μmol m^−2^ s^−1^, which must be generated through LEF.

In a PEPCK C_2_+C_4_ photosynthetic subtype (Supplementary Fig. S4C), the C_4_ CCM results in the same export of CO_2_ to the BS as in the NADP-ME and NAD-ME C_2_+C_4_ photosynthetic subtypes. However, although MDH is present in the M, PEPCK activity in the BS diverts OAA produced by PEPC in the M, making OAA unavailable for MDH activity in the M, which is consequently zero. Furthermore, because MDH activity is zero, the CCM does not export reducing power to the BS, and the NADPH must be supplied through LEF in the BS.

#### Simulation 3.2. Dynamic simulations

**Fig. 3.**
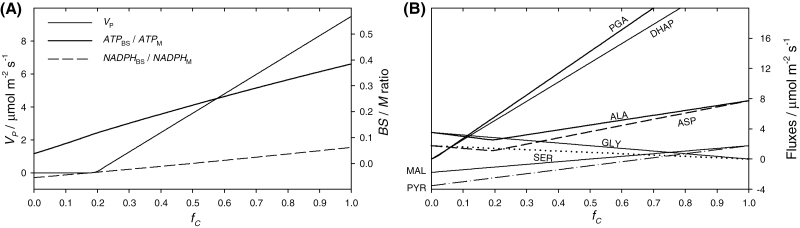
Simulation 3.2. From C_2_ to C_4_ photosynthesis. The BS partitioning of Rubisco activity (*f*_C_) was varied between 0 and 1. Panel (**A**) shows the rate of PEPC activity (*V*_P_) that resulted in no net CO_2_ flux across the BS–M interface, and the resultant ATP and NADPH partitioning. Panel (**B**) shows the corresponding metabolite fluxes. Inputs are shown in Table 3.

Intermediate states of ‘C_4_ness’, represented by intermediate degrees of *f*_C_ and *V*_P_, were explored to simulate the transition from C_2_ photosynthesis to C_4_ photosynthesis. *f*_C_ was incrementally varied, and, at each *f*_C_ level, *V*_P_ was iteratively fitted so that the CO_2_ leak rate (*L*) remained zero (Supplementary Equation S34). The resultant *V*_P_ as a function of *f*_C_ is plotted in Fig. 3A. Values of *V*_P_ above the curve will result in an effective CCM. Values of *V*_P_ below the curve are insufficient to sustain Rubisco fixation, which will drive a net influx of CO_2_ into the BS. For *f*_C_ < 0.2, Rubisco fixation in the BS is supplied by the C_2_ shuttle, meaning the predicted *V*_P_ is zero. Moving Rubisco to the BS causes the locality of ATP and NADPH demand to change (Fig. 3A). Reducing power demand in the BS is low and is only used by the PCO cycle by setting *f*_PR_ at zero. This requires PGA to diffuse out of the BS and in an opposite flux of DHAP, which is lower than that of PGA by a value equal to CS. In addition, ALA and ASP are used by the CCM to bypass MDH in the M. The concave trend of ALA and ASP fluxes reflects a decreasing recruitment for the C_2_ shuttle at low *f*_C_ and an increasing recruitment for the CCM at high *f*_C_. GLY and SER are recruited only by the C_2_ shuttle and decrease to zero with *f*_C_. MAL and PYR are used by the C_4_ cycle and their fluxes increase linearly with *f*_C_.

### Simulation 4. C_4_ photosynthetic subtypes

Here, Rubisco and GDC are completely compartmentalized to the BS and *V*_P_ exceeds *V*_C_.

#### Simulation 4.1. Operational conditions

##### Simulation 4.1.1.

Outputs for a typical NADP-ME subtype are shown in Supplementary Fig. S5A. The activity of the CCM is strong enough to exceed Rubisco carbon fixation in the BS and a positive CO_2_ leakage out of the BS. PR is mainly located in the M and metabolite traffic is up to five-times that of gross assimilation (*GA*). The net effect of the C_2_ shuttle (as *R*), which depends on Rubisco oxygenating activity in the M, is zero. The CCM supplies all *NADPH*_BS_ (6.54 μmol m^−2^ s^−1^), no LEF is required in the BS, and there is excess OAA that is not reacted with by MDH and is subsequently transaminated (*T*).

##### Simulation 4.1.2.

Supplementary Fig. S5B shows a typical NADP-ME subtype with engagement of PEPCK. PEPCK activity regenerates part of the PEP required by PEPC, driving a positive PEP flux out of the BS, which reduces the activity of PPDK in the M. PEPCK consumes half the ATP of PPDK, resulting in a 4% lower *ATP*/*GA* than for the NADP-ME subtype. However, PEPCK activity is generally low, all *NADPH*_BS_ is supplied by the CCM, and no LEF is required in the BS.

##### Simulation 4.1.3.

Supplementary Fig. S5C shows a typical NAD-ME C_4_ photosynthetic subtype with no engagement of PEPCK. The CCM exports CO_2_ at the same rate as the NADP-ME subtype; however, MDH activity in M is zero, all OAA is transaminated, and the NADPH demand in the BS has to be generated in the BS through LEF.

##### Simulation 4.1.4.

Supplementary Fig. S5D shows a background NADP-ME metabolism using PEPCK as a sole decarboxylase (*r*_PEPCK_ = 1). The CCM exports CO_2_ at the same rate as the other subtypes. Because MDH is present in the M, the CCM would export reducing power to the BS if *r*_PEPCK_ < 1, but, here, PEPCK activity in the BS diverts OAA produced by PEPC in the M, making OAA unavailable for MDH activity in the M, which is consequently zero. Furthermore, because MDH activity is zero, the CCM does not export reducing power to the BS, and the NADPH must be supplied through LEF in the BS.

##### Simulation 4.1.5.

Supplementary Fig. S5E shows SMA output for a background NAP-ME metabolism using PEPCK as a sole decarboxylase (*r*_PEPCK_ = 1). The CCM exports CO_2_ at the same rate as the other subtypes. Here, MDH in M is not operational, and the CCM does not have the potential to export reducing power to the BS.

#### Simulation 4.2. Dynamic simulations (C_4_ plasticity mechanisms)

For the next four simulations, *r*_O/C_ was set at a typical C_4_ value of 0.05 (Bellasio *et al.*, 2014); *V*_P_ was set at 10.85 estimated after von Caemmerer (2000); and gradual transitions of *f*_CS_, *f*_PR_, *r*_PEPCK_, and *f*_PPDK_ were simulated by calculating the SMA for 21 discrete values of *f*_CS_, *f*_PR_, *r*_PEPCK_ , and *f*_PPDK_ between 0 and 1. Other model inputs are listed in Table 3.

##### Simulation 4.2.1. Partitioning PR.

**Fig. 4.**
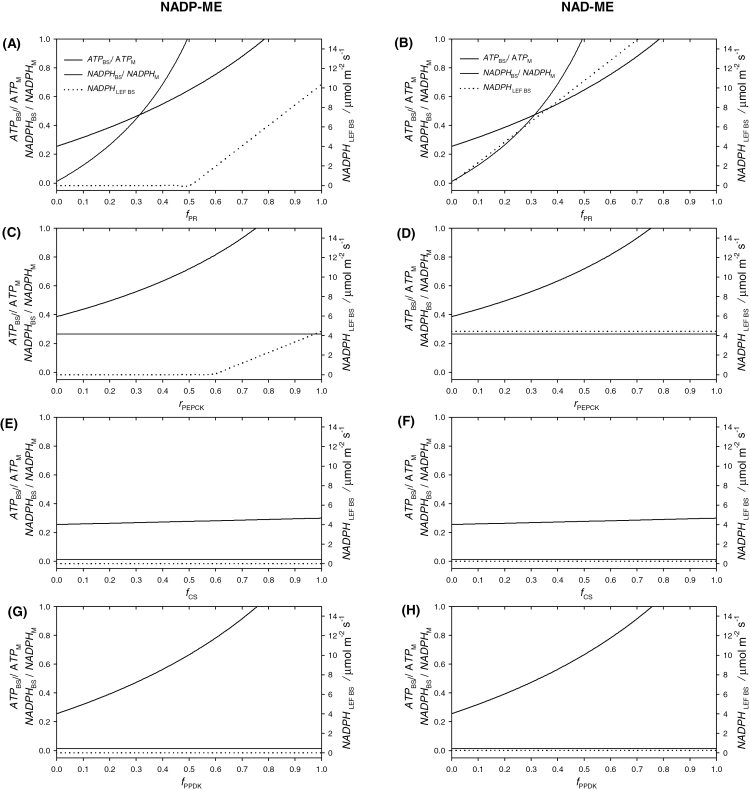
Simulation 4.2. SMA simulations showing the effect of varying the BS engagement in PR (**A, B**), PEPCK activity (**C, D**), the BS engagement in CS (**E, F**), the BS fraction of PPDK activity (**G, H**), on the partitioning of ATP demand (thick lines), the partitioning of NADPH demand (thin lines), and on the rate of NADPH produced by LEF in the BS (dotted lines, right axes) in a background NADP-ME (left) or NAD-ME (right) subtype. Inputs are shown in Table 3.

The fraction of PGA reduced in the BS was manipulated through the input parameter *f*_PR_, while *r*_PEPCK_, *f*_PPDK_, and *f*_CS_ were kept at zero. An increase in *f*_PR_ caused an increase in the ATP and NADPH demand in the BS, which occurred in both NADP-ME and NAD-ME subtypes (Fig. 4A, B). The NADPH demand to be supplied by LEF in the BS followed different trends. In the NADP-ME subtype, with an *f*_PR_ of up to 0.5, the NADPH demand for PR was met by the CCM through the MAL shuttle, and the resultant NADPH demand through LEF was zero. Additional levels of PR (*f*_PR_ > 0.5) required the engagement of LEF in the BS. In the NAD-ME subtype, the demand for LEF in the BS increased linearly for *f*_PR_ > 0.

##### Simulation 4.2.2. Manipulating PEPCK activity.

The engagement of PEPCK was manipulated through the input parameter *r*_PEPCK_. *f*_PR_ was set at 0.25 to highlight differences between NADP-ME and NAD-ME subtypes, while *f*_PPDK_ and *f*_CS_ were kept at zero (Table 3). An increase in *r*_PEPCK_ increased the ATP demand in the BS in both the NADP-ME and NAD-ME subtypes (Fig. 4C, D), but the partitioning of NADPH demand was unaffected. The NADPH demand to be supplied by LEF in the BS, however, followed different trends. In the NADP-ME subtype, with an *r*_PEPCK_ of up to 0.6, the NADPH demand through LEF was zero, and increased linearly up to 4.5 μmol m^−2^ s^−1^ for *r*_PEPCK_ > 0.6. In the NAD-ME subtype, the NADPH demand to be supplied by LEF was constant at 4.5 μmol m^−2^ s^−1^, regardless of *r*_PEPCK_.

##### Simulation 4.2.3. Partitioning CS.

In the simulation, the BS fraction of CS was manipulated to increase through the input parameter *f*_CS_, while *r*_PEPCK_, *f*_PPDK_, and *f*_PR_ were kept at zero. Increasing *f*_CS_ determined a marginal increase in *ATP*_BS_/*ATP*_M_ from 0.26 to 0.29 in both the NADP-ME and NAD-ME subtypes, while the NADPH demand was unaffected (Fig. 4E, F).

##### Simulation 4.2.4. Effect of PPDK engagement in the BS.

The effect of PPDK engagement in the BS was studied by manipulating the input parameter *f*_PPDK_, while *r*_PEPCK_, *f*_CS_, and *f*_PR_ were kept at zero. The ATP demand in the BS increased substantially, while the NADPH demand was unaffected in both the NADP-ME and NAD-ME subtypes (Fig. 4G, H).

## Discussion

Flux-balance analysis models are constraint-based models in which steady state fluxes are predicted in a metabolic network by applying mass-balance constraints based on reaction stoichiometry (Sweetlove and Ratcliffe, 2011). Lately the complexity of models has grown to embrace a suite of photosynthetic processes (Laisk *et al.*, 2009; Wang *et al.*, 2014*a*; Wang *et al.*, 2014*b*) and reconstruct genome-wide metabolism (Dal’Molin *et al.*, 2010; Saha *et al.*, 2011). Given this complexity, these models are only available for a few well-studied species and modifying them requires considerable coding effort, meaning they are not ideal for studying bio-manipulation or testing hypotheses. The metabolic model developed by Bellasio and Griffiths (2014*c*) facilitated straightforward modifications and changes to metabolic pathways. For instance, the model computed the effects of partitioning biochemical work between the BS and M on the locality of ATP and NADPH demand in photosynthesizing C_4_ leaves, and was modified to support the interpretation of biochemical, gas exchang,e and transcriptomic data in engineered sugarcane (McQualter *et al.*, 2016). Based on these theoretical underpinnings, the SMA was developed to account for the interactions between C_2_ and C_4_ cycles, including the fluxes associated with amino group rebalancing, recently described in Mallmann *et al.* (2014), and their effect on NADPH and ATP availability. The SMA integrates assimilatory metabolism and energetics as well as calculating key reaction rates, metabolite traffic fluxes, and ATP and NADPH requirements in the M and BS when the locality of GDC (as *f*_GDC_) and Rubisco (as *f*_O_, and *f*_C_) activity, leaf-level Rubisco rates of carboxylation and oxygenation (as *r*_O/C_), and PEP engagement (as *V*_P_) are known. While previously published models are tailored for a particular species or a discrete photosynthetic type that is fixed *a priori*, the SMA allows rates to vary continuously between boundaries defined by what is biochemically feasible. The SMA is designed for integration with existing biochemical models. However, these models rely on assumptions that are specific to a particular photosynthetic type and, as such, they were not included in the SMA at this stage. The SMA is based upon logical constraints and well-established reaction stoichiometry, and is, consequently, of general use. Compared with other recent flux-balance models [e.g. Dal’Molin *et al.* (2010)], the SMA has several distinctive features. It is focused on assimilation; it generalizes all pathways of carbon assimilation in a single tool, except CAM, which requires an explicit temporal dynamic (Owen and Griffiths, 2013); it allows straightforward modification by the user; and it explicitly accounts for NADPH and ATP demand, allowing the study of the effect of biochemical regulation on energetics.

The following discussion refers to the simulations described in simulations 4.2.1–4.2.4 (C_4_ plasticity mechanisms, Fig. 4), which are of particular interest for this special issue. Possible mechanisms that C_4_ plants can exploit to stabilize their energy and redox state were recently reviewed (Stitt and Zhu, 2014). Reversible reactions linking PGA and triose phosphates, the reversibility of the PPDK reaction, the inter-conversion of PEP and PGA, and the transient build-up and utilization of PGA and triose phosphate require explicit temporal dynamics to be modelled and cannot be addressed by the SMA. Nevertheless, the futile cycles involved probably lower the biochemical efficiency of assimilation and are likely to be downregulated under steady state conditions. The other processes reviewed by Stitt and Zhu (2014) are shown in the simulation results. Specifically, the rate of ASP/MAL decarboxylation was mechanistically linked to reducing power requirement in the BS through Supplementary Equation S21, and can be followed in Supplementary file 2 (workbook cell O14); CO_2_ leakage and leakiness are calculated by balancing all CO_2_ fluxes in and out of the BS (Supplementary Equation S34) and can be followed in Supplementary file 2 (workbook cells O19 and O6, respectively); the exchange of triose phosphate and PGA and the effect of switching decarboxylase (ME versus PEPCK) are covered in simulations 4.2.1 and 4.2.2. In addition to the list of Stitt and Zhu (2014), here I investigated a possible role of CS by manipulating *f*_PR_ (simulation 4.2.3), and I suggest a possible role for PPDK in the BS, which was investigated by manipulating *f*_PPDK_ (simulation 4.2.4). The simulations were carried out as a single factor analysis in which only one input was varied at a time (Table 3). This approach has the benefit of allowing a comparison of the effectiveness of different plasticity mechanisms. Combining effects, however, is also straightforward: when multiple inputs are manipulated, the total effect is the sum of single effects. The four simulations are discussed separately.

### Partitioning PR and regulation of NADPH demand

When BS engagement in PR was manipulated to increase, ATP and NADPH demand in the BS also increased. In particular, in NADP–ME subtypes above a *f*_PR_ of 0.5, there was a substantial demand for NADPH through LEF in the BS. While ATP is produced by BS photophosphorylation in both NADP-ME and NAD-ME plants, BS chloroplasts in NADP-ME plants are generally not able to oxidize water and produce substantial amounts of NADPH. In NADP-ME plants, high levels of *f*_PR_ are therefore unlikely, consistent with the observation that, in maize, PGA is mainly reduced in the M (Friso *et al.*, 2010). Confining O_2_ production to M chloroplasts, away from Rubisco carboxylating sites, contributes to photorespiration suppression (Majeran and van Wijk, 2009), but imposes functional trade-offs. The inability to sustain high levels of PR in the BS requires PGA to diffuse to M cells, and DHAP to return to BS cells in order to complete the RPP cycle (Fig. 1). This imposes substantial traffic across the M–BS interface that is five-times that of *GA* (Supplementary Fig. S5A–E). For this traffic, high BS conductance would be beneficial, but imposes high levels of CO_2_ retrodiffusion, and limits the effectiveness of the CCM (Bellasio and Griffiths, 2014*a*). Because of this trade-off, BS conductance is thought to be tightly regulated (Kromdijk *et al.*, 2014), and was observed to scale with assimilation to optimize the efficiency of the CCM (Ubierna *et al.*, 2013; Bellasio and Griffiths, 2014*b*; von Caemmerer and Furbank, 2016). The available evidence, however, is indirect and further investigation is required.

### Manipulating PEPCK activity

While ATP is directly used in PEPCK reactions, PEPCK activity does not directly require NADPH, as shown by the plot of NADPH demand in Fig. 4C. The effect on NADPH demand through LEF is therefore indirect and depends on the subtype (NADP-ME or NAD-ME). NADP-ME subtypes have the potential to export reducing power to the BS through MDH activity in the M: when PEPCK activity increases, increasing levels of OAA are required, which are not available for MDH in the M and cannot be used to shuttle reducing power from the M to the BS through MAL. In other words, the NADPH potentially available through the CCM decreases. Ultimately, when PEPCK is fully engaged, there is no surplus of OAA available for exporting NAPDH to the BS and all NADPH demand in the BS must be met by LEF. In NAD-ME subtypes, which do not have the capacity to export reducing power, all NADPH demand has to be met by LEF. When PEPCK is the only decarboxylating enzyme, irrespective of the presence of MDH in the M (that is, in both NAD-ME and NADP-ME subtypes), no reducing power can be exported to the BS (Fig. 2C, D). Wang *et al.* (2014*a*) noted considerable variability in PEPCK engagement. For instance, in the NADP-ME subtypes there is a gradient from *Sorghum bicolor* with virtually no PEPCK engagement, through low engagement in *Flaveria* species, intermediate engagement in maize and sugarcane (*Saccharum* species), to virtually complete engagement in the atypical C_4_*Alloteropsis semialata* (Gutierrez *et al.*, 1974; Ueno and Sentoku, 2006; Wang *et al.*, 2014*a*; Lundgren *et al.*, 2016; Dunning *et al.*, in review). Similarly, in NAD-ME subtypes the gradient spans *Panicum* species with virtually no PEPCK engagement, *Cleome* C_4_ species with intermediate PEPCK activity, and *Spartina maritima* where PEPCK engagement is virtually complete (Gowik and Westhoff, 2011; Bellasio and Griffiths, 2014*c*; Lundgren *et al.*, 2016). Regardless of how this variability is classified (Furbank, 2011; Wang *et al.*, 2014*a*; Koteyeva *et al.*, 2015), the SMA allows rates to vary continuously between boundaries set by what is biochemically realistic.

Given that PEPCK regenerates PEP with half the ATP required by PPDK, moderate levels of PEPCK engagement could potentially increase the biochemical efficiency of assimilation (compare *ATP*_TOT_/*GA* in Supplementary Fig. S5A with Fig. S5D). The rapid regulation of PPDK (Leegood and Walker, 1999) could contribute to the flexibility and efficiency of the CCM, supported by the observation that redundant decarboxylating pathways appeared multiple times at late evolutionary stages (Christin and Osborne, 2014). At high levels of PEPCK engagement (Supplementary Fig. S5D, E), the *ATP*/*GA* predicted is substantially lower than for the other photosynthetic subtypes. However, biochemical efficiency may be reduced by the need to hydrolyse part of the newly synthetized PEP to drive the PEPCK reaction, which is close to thermodynamic equilibrium and may be too slow to support physiological decarboxylation rates (Huber and Edwards, 1975). Clarification is still required, and there may be interspecific variability (Smith and Woolhouse, 1983); however, circumstantial evidence gained in comparative experiments have shown lower quantum efficiency of PEPCK plants [Furbank (2011) and references therein].

PEPCK is required for the CCM to work, and when PEPCK is the only decarboxylating enzyme it is likely to be modulated solely by the requirements of the CCM. As a consequence, in PEPCK plants, PEPCK activity cannot contribute to fine-tuning ATP demand, and hence the biochemical plasticity of the CCM is lower. The additional ATP used by PEPCK can therefore be considered part of the minimum ATP demand in the BS, *ATP*_BSMIN_ = *RuP*_phospBS_ + *PEPCK + V*_OBS_ + *R*, corresponding to a minimum ATP demand partitioning ratio of $\frac{ATP_{\text{BSMIN}}}{ATP_{\text{M}}}\approx 1,$ which is approximately three times that of other C_4_ subtypes (see below). Although PEPCK plants have numerous chloroplasts in the BS (Dengler and Nelson, 1999; Bellasio and Lundgren, 2016), limiting environmental conditions (e.g. dim diffuse sky light) may prevent the BS from supplying *ATP*_BSMIN_. In these conditions ATP could be synthetized by mitochondria, thus countering the lower biochemical plasticity of the CCM with additional flexibility in ATP generation. The isolated BS of some PEPCK plants can, under ATP starvation, convert MAL-derived NADH into ATP through mitochondrial oxidation of NADH (Carnal *et al.*, 1993). This process is less energy efficient than photophosphorylation (Buckley and Adams, 2011; Kramer and Evans, 2011), and may contribute to the generation of *ATP*_BSMIN_ only when illumination of the BS chloroplast is insufficient.

### Partitioning CS

The main photosynthetic products in C_4_ species are starch and sucrose. Both can be synthesized in the M and BS (Majeran and van Wijk, 2009; Friso *et al.*, 2010), but sucrose is preferentially synthesized in the M, while starch disproportionately accumulates in the BS (Furbank *et al.*, 1985; Lunn and Furbank, 1997). It has been observed that accumulation of sucrose in the leaf does not directly influence the partitioning between sucrose and starch (Lunn and Hatch, 1995), apparently discounting a role for starch synthesis as a sink for carbon overspill when sucrose synthesis is inhibited by sucrose build-up (Lunn and Hatch, 1995). Experiments conducted with *Panicum* species examined the effect of illumination on the distribution of sucrose–phosphate synthase activity between the M and BS, showing contrasting activation patterns between subtypes (Ohsugi and Huber, 1987). Here, a role of energetics is unlikely given the overall negligible ATP cost of CS. Alternative explanations involve an effect of the ratio of PGA to inorganic phosphate on the activity of ADP-glucose pyrophosphorylase [Stitt *et al.*, 1987; Lunn and Furbank, 1997; for further considerations see Ap Rees (1987), Furbank (1992), Leegood and Walker (1999), Zeeman *et al.* (2007), Kotting *et al.* (2010), and Weise *et al.* (2011)].

### Effect of PPDK engagement in the BS and fast regulation of ATP demand

PPDK was shown to be present and active in the BS (Aoyagi and Nakamoto, 1985; Friso *et al.*, 2010), although its elusive role is generally not included in textbook descriptions of C_4_ photosynthesis. The engagement of PPDK increases ATP demand in the BS in the same way as PR, as both processes require two ATP per catalytic turnover. However, PPDK does not require NADPH, whereas PR does. I propose that the tight regulation of PR and PPDK activities (and, in some cases, PEPCK, see above) contribute to fine-tuning ATP and NADPH demand in the BS in response to illumination of the BS chloroplast. Light availability in the BS is determined by anatomy and changes dynamically according to light intensity and quality. The locality of ATP production is therefore largely independent of metabolic control [Bellasio and Lundgren (2016) and references therein]. Local ATP imbalances cannot be rebalanced by ATP diffusion because maintaining ATP concentration and a high ATP to ADP ratio in each cell compartment is critical. The only way to balance supply and demand in each compartment is to regulate the locality of ATP demand (Evans *et al.*, 2007; Bellasio and Griffiths, 2014*c*). For instance, blue-rich diffuse sky radiation is strongly absorbed in the superficial M and results in preferential ATP production in the M (Evans *et al.*, 2007). In these conditions BS activity can be downregulated, and countered by a proportional increase in M activity, to maintain assimilation and biochemical efficiency. However, operating the RPP, the C_2_, and PCO cycles in the BS impose a limit, and a threshold of ATP demand in the BS (*ATP*_BSMIN_ = *RuP*_phospBS_ + *V*_OBS_ + *R*), corresponding to an ATP partitioning ratio of $\frac{ATP_{\text{BSMIN}}}{ATP_{\text{M}}}\approx 0.3.$ If ATP supply in the BS is lower than the minimum demand, C_4_ photosynthesis ceases, leading to stunted phenotypes (Bellasio and Griffiths, 2014*c*; McQualter *et al.*, 2016). More penetrating light (e.g. red light or direct sunlight) may drive higher ATP synthesis in the BS. In this case, PR and PEPCK may be upregulated, but are limited by the amount of NADPH available in the BS through the MAL shuttle or through LEF, as discussed above. PPDK may be engaged to take advantage of additional ATP, which may be made available under transient exposure to even more penetrating light qualities (e.g. green-enriched canopy light).

## Conclusion

A SMA has been developed as a modelling framework based solely on mass-balance constraints, which generalizes all pathways of assimilation (except CAM) in a single tool. A range of examples detailed the energetics and metabolite fluxes involved in the gradual activation of the C_2_ shuttle and the CCM along a spectrum of photosynthetic subtypes from C_3_ to C_4_ photosynthesis. This knowledge is important for basic and applied research, to support advanced breeding techniques, or to study natural variability of biochemical traits. For instance, by providing quantitative estimates for fluxes and energy requirements in the BS and M, it is possible to set realistic targets for bioengineering projects. Future work will integrate biochemical models to allow the SMA to respond directly to environmental variables.

## Availability

SMA is coded in an Excel workbook that is freely available to download. Macros are avoided.

## Supplementary data

Supplementary data are available at *JXB* online.

File 1. SMA development and equations S1–S34; simulation of static scenarios under physiological operational conditions: Table S1 and Figures S1, S3–S5E; simulation 1.2, a dynamic scenario for C_3_ photosynthesis: Figure S2.

File 2. Excel workbook coding the SMA.

## Supplementary Material

Supplementary_Table_S1_Figures_S1_S5

Supplementary_Data_Model
