# Supplementary material for: A generalized stoichiometric model of C3, C2, C2+C4, and C4 photosynthetic metabolism
Source: J Exp Bot. 2016 Aug 17;68(2):269–82. doi: 10.1093/jxb/erw303 (PMC5853385; doi:10.1093/jxb/erw303)
Supplement: Supplementary_Table_S1_Figures_S1_S5 [file erw303_suppl_supplementary_table_s1_figures_s1_s5.pdf]

A generalised stoichiometric model of C_3, C_2, C_2+C_4, and C_4 photosynthetic metabolism

SMA Development

1. Leaf–level reaction rates, NADPH and ATP demand.

Leaf metabolism is defined in terms of gross assimilation *GA*, and Rubisco rates of carboxylation and oxygenation, V_O, and V_C. These may be inputted directly if available (see *Parameterisation*, below) or calculated from net assimilation (*A*), respiration in the light (R_{LIGHT}), and oxygenation / carboxylation ratio $r_{O/C}$ using these equations:

$$GA = A + R_{\mathrm{LIGHT}}, \quad (1)$$

$$V_C = \frac{GA}{1 - 0.5\, r_{\mathrm{O/C}}}, \quad (2)$$

$$V_{\mathrm{O}} = V_{\mathrm{C}}\, r_{\mathrm{O/C}}\ . \quad (3)$$

Based on these, the rates of key processes such as PGA reduction (PR), RuBP regeneration, Reductive pentose phosphate cycle (RPP) turnover rate and carbohydrate synthesis (CS) are calculated at leaf–level and define the overall NADPH and ATP demand.

Knowing that the carboxylating activity of Rubisco generates PGA at a rate of $2V_C$, the oxygenating activity of Rubisco produces PGA at a rate of V_O, glycolate recycling regenerates PGA at a rate of $\frac{1}{2}V_O$, in the SMA respiration is supplied by newly–synthesised PGA (Stutz *et al.*, 2014) and consumes PGA at a rate of $1/3R_{LIGHT}$ (where 1/3 is the stoichiometric conversion between CO_2 and trioses), the total rate of PGA reduction (PR_{TOT}) is:

$$PR_{\mathrm{TOT}} = 2V_{\mathrm{C}} + \frac{3}{2}V_{\mathrm{O}} - \frac{1}{3}R_{\mathrm{LIGHT}}. \quad (4)$$

The rate of RuP phosphorylation required to supply Rubisco is:

$$RuP_{\mathrm{phosp}} = V_{\mathrm{C}} + V_{\mathrm{O}}. \quad (5)$$

In the SMA the RPP cycle operates uniquely to regenerate RuP. The rate of DHAP entering RPP is:

$$DHAP_{\mathrm{RPP}} = \frac{5}{3} RuP_{\mathrm{phosp}}, \quad (6)$$

where 5/3 is the ratio between the number of carbon atoms in RuP (C_5) and DHAP (C_3).

In the SMA the final product of photosynthesis is a general compound called 'carbohydrates' with the stoichiometry of trioses; carbohydrate synthesis (CS) is supplied by DHAP. The total leaf–level CS rate (CS_{TOT}) results from subtracting the rate of DHAP used to regenerate RuP ($DHAP_{\mathrm{RPP}}$) from the total DHAP production rate (corresponding to PR_{TOT}, Eqn 4):

$$CS_{\mathrm{TOT}} = PR_{\mathrm{TOT}} - DHAP_{\mathrm{RPP}}. \quad (7)$$

The total glycine decarboxylation rate GDC_{TOT}, expressed with the stoichiometry of glycine consumption, is equivalent to V_{O}.

The total leaf–level NADPH demand ($NADPH_{\mathrm{TOT}}$) can be calculated knowing that PGA reduction consumes 1 NADPH per PGA, and ½NADPH is consumed per glycolate recycled [½NADH is produced by glycine decarboxylase, ½NADH is consumed by hydroxypyruvate reductase and one ferredoxin (equivalent to ½NADPH) is consumed by GOGAT] as:

$$NADPH_{\mathrm{TOT}} = PR_{\mathrm{TOT}} + \frac{1}{2} V_{\mathrm{O}}. \quad (8)$$

The ATP cost of CS was fixed at ½ATP. This ATP cost is consistent with starch synthesis and phloem–loaded sucrose, considering the stoichiometry of 1 H^+ / sucrose of the sucrose symporter and 1 ATP / H^+ of the plasma membrane H^+ – ATPase energising sucrose uptake. Knowing that PGA reduction consumes 1 ATP per PGA, RuBP regeneration consumes 1 ATP per RuP, the PCO cycle consumes 1 ATP per glycolate (½ATP/glycolate is consumed by glutamine synthetase, ½ATP/glycolate is consumed by glycerate kinase), CS consumes ½ATP per triose, and PEP regeneration consumes 1 ATP per PEPCK catalytic event or 2 ATP per PPDK catalytic event, the total leaf–level ATP demand (ATP_{TOT}) is:

$$ATP_{\mathrm{TOT}} = PR_{\mathrm{TOT}} + RuP_{\mathrm{phosp}} + V_{\mathrm{O}} + \frac{1}{2} CS_{\mathrm{TOT}} + PEPCK + 2PPDK. \quad (9)$$

Compared with the formulation of von Caemmerer (2000), Eqn 9 separates the ATP demand for PEPCK and PPDK, includes the ATP demand for CS, and subtracts the PGA consumed by respiration in the light (Eqn 4).

2. The activity of the CCM

The activity of the CCM is defined in terms of PEPC reaction rate (V_P). The CCM is operational when V_P exceeds V_C (overcycling) and a positive CO_2 leakage (L) is established. The sub–type of CCM (Table 3) is determined by switching MDH in the M on or off (see Eqn 19) and by varying the activity of PEPCK. This logic prioritises the definition of PEPCK activity over balancing reducing power and transamination, based on the recognition that while *PEPCK* is tightly regulated by ATP availability (Leegood and Walker, 1999), aminotransferases operate fast equilibrium interconversions (Leegood and Walker, 1999; Wang *et al.*, 2014).

V_P is a direct input to the SMA (Workbook cell C15). *PEPCK*, which is generally expressed as a fraction of V_P (Koteyeva *et al.*, 2015), in the SMA is specified through the input factor r_{PEPCK}, and PEPCK reaction rate (*PEPCK*) is calculated as:

$$PEPCK = V_{\mathrm{P}}\, r_{\mathrm{PEPCK}} \,. \qquad 10$$

When r_{PEPCK}=1, PECK is the only decarboxylating enzyme. In these conditions PEPCK operates at its upper limit, set by the availability of the substrate OAA in the BS (Fig. 1).

The fate of PEP generated by PEPCK activity is equivocal (Smith and Woolhouse, 1983). The most energy efficient option is that PEP diffuses to the M and supplies PEPC, thus reducing the PEP demand on PPDK. Under these circumstances the total PPDK reaction rate ($PPDK_{\mathrm{TOT}}$) is:

$$PPDK_{\mathrm{TOT}} = V_{\mathrm{P}} - PEPCK. \qquad 11$$

If, instead, some of the PEP produced by PEPCK in the BS is hydrolysed (see *Discussion*), an additional term needs to be incorporated into the right–hand–side of Eqn 11 (and of Eqn 28 and 33).

3. The contribution of the M and BS

The role of the BS in photosynthetic metabolism varies depending on the photosynthetic type and on environmental inputs. In the SMA, the contribution of the BS is determined by partitioning R_{LIGHT}, V_O, V_C, *GDC*, CS_{TOT}, and $PPDK_{\mathrm{TOT}}$, derived above, between the BS and M. The general BS partitioning equation is:

$$Activity_{\mathrm{BS}} = Activity_{\mathrm{TOT}} \cdot f_{BS/TOT} \,, \qquad 12$$

while, the activity in the M is the remainder of the total.

V_O, V_C, GDC_{TOT}, R_{LIGHT}, CS_{TOT}, PR_{TOT}, and $PPDK$ are partitioned through the inputs f_O, f_C, f_{GDC}, and f_{RLIGHT}, f_{CS}, f_{PR} and f_{PPDK} respectively, (Table 2).

The BS fraction of $DHAP_{RPP}$, and RuP_{phosp} are calculated in analogy to Eqn 5 and 6 as:

$$RuP_{\text{phosp BS}} = V_{\text{C BS}} + V_{\text{O BS}} \ , \quad (13)$$

and

$$DHAP_{\text{Rpp BS}} = \frac{5}{3} RuP_{\text{phosp BS}} \ . \quad (14)$$

The ATP demand in the BS (see also description of Eqn 9), is:

$$ATP_{\text{BS}} = PR_{\text{BS}} + RuP_{\text{phosp BS}} + V_{\text{O BS}} + \frac{1}{2}\ CS_{\text{BS}} + PEPCK + 2PPDK_{\text{BS}} + R\ , \quad (15)$$

where R is the rate of CO_2 and NH_3 release in the BS associated with the operation of the C_2 shuttle, which in Eqn 15 accounts for the additional ATP cost of NH_3 recapture through GS/GOGAT, and can be expressed as:

$$R = \frac{1}{2}(GDC_{\text{BS}} - V_{\text{O BS}}) = \frac{1}{2}\left(V_{\text{O M}} - GDC_{\text{M}}\right)_{\text{BS}} . \quad (16)$$

Similarly, the ATP demand in the M (see also description of Eqn 9 and Eqn 15) is calculated as:

$$ATP_{\text{M}} = 2\ PPDK_{\text{M}} + RuP_{\text{phosp M}} + V_{\text{O M}} + \frac{1}{2}\ CS_{\text{M}} + PR_{\text{M}} - R. \quad (17)$$

4. Balancing NADPH demand in the BS

To generalise NAD–ME and NADP–ME subtypes, NADPH and NADH are considered equivalent or convertible and cells are assumed to be decompartmentalised.

The NADPH demand in the BS (see description of Eqn 8) is:

$$NADPH_{\text{BS}} = PR_{\text{BS}} + \frac{1}{2} V_{\text{O BS}} - R, \quad (18)$$

here R (Eqn 16) accounts for the NADH generated in the BS by GDC resulting from decarboxylating GLY imported from the M through the C_2 shuttle.

In NADP–ME plants (with or without partial PEPCK engagement) MDH is active in the M. MDH activity (MDH_M) results in the transfer of NADPH from M to BS. In the SMA

MDH_M can be limited either by the reducing power demand in the BS, or by substrate availability. The maximum rate of OAA supply to MDH in M is V_P-*PEPCK* (the total OAA production rate V_P, is subtracted the fraction of OAA required for PEPCK activity in the BS). MDH_M can therefore be expressed as:

$$MDH_{\mathrm{M}} = \min(NADPH_{\mathrm{BS}}, V_{\mathrm{P}} - PEPCK). \qquad 19$$

In NAD–ME subtypes MDH in M is not active resulting in no net reducing power export mediated by the CCM. MDH_M may be simply set at zero (workbook cell K24).

The NADPH demand in the BS may exceed the supply by MDH_M, particularly in NAD–ME types where MDH_M=0. The fraction of NADPH demand in the BS not supplied by the CCM, must be supplied by linear electron flow (LEF) in the BS. The NADPH demand through LEF is:

$$NADPH_{\mathrm{LEF\,BS}} = \max[NADPH_{\mathrm{BS}} - MDH_{\mathrm{M}}, 0]\,. \qquad 20$$

In NAD–ME subtypes all of the OAA generated by PEPC is transaminated. In NADP–ME subtypes transamination mediates a redundant mechanism delivering CO_2 to the BS, which allows the CCM to operate independently of the NADPH demand in the BS [see (Bellasio and Griffiths, 2014) and references therein]. Transamination rate (*T*) is calculated as the fraction of OAA not reacted by MDH in the M:

$$T = V_P - MDH_M. \qquad 21$$

The rate of malate dehydrogenase in the BS (MDH_{BS}) is:

$$MDH_{\mathrm{BS}} = T - PEPCK. \qquad 22$$

The malic enzyme (ME) activity in the BS is:

$$ME = MDH_{\mathrm{M}} + MDH_{\mathrm{BS}}. \qquad 23$$

And, finally, the rate of NADPH demand through LEF in the M is:

$$NADPH_{\mathrm{LEF\,M}} = PR_{\mathrm{M}} + MDH_{\mathrm{M}} + \frac{1}{2}V_{\mathrm{O\,M}} + R. \qquad 24$$

Eqn 20–24 are of general use for NAD–ME and NADP–ME photosynthetic subtypes, with or without PEPCK engagement.

In the calculation of NADPH and ATP demand the SMA only accounts for assimilatory processes, for consistency with gas exchange data analysis (Bellasio *et al.*, 2016; Bellasio *et al.*, 2014). For the same reason any ATP and NADH residual imbalances produced during respiration in the light are absent from the calculations, and assumed to be entirely consumed by basal metabolism. This is supported by the analysis of Buckley and Adams (2011) who computed the NADPH excess caused by basal metabolism, and proposed that this would be dissipated by mitochondrial alternative oxidases. If this were not the case, then that excess could, in principle, be added to the SMA to refine the estimate for $NADPH_{\mathrm{LEF}}$, but it is small (0.1–0.3 µmol m^{-2} s^{-1}) in comparison with assimilatory requirements. Similarly, any possible NADPH demand for mitochondrial ATP generation could be separately calculated and then integrated in the SMA.

5. Metabolite Fluxes between the M and BS, and amino–group balancing

Sharing biochemical work between the BS and M results in metabolite traffic. The SMA calculates the fluxes of MAL, PEP, PYR, ASP, ALA, PGA, DHAP, GLY, SER, as well as unbound CO_2 across the M–BS interface (Fig. 1). The fluxes between the M and BS can be calculated either by M or BS mass balance, the simpler of the two equivalent alternatives is described. Fluxes are considered positive when occurring in the direction of the arrow, and vice versa.

The flux of DHAP from M to BS is calculated from DHAP mass balance in the M as:

$$DHAP_{\mathrm{M\rightarrow BS}} = PR_{\mathrm{M}} - CS_{\mathrm{M}} - DHAP_{\mathrm{RPP\ M}}. \quad 25$$

The flux of PGA from BS to M is calculated from PGA mass balance in the BS as:

$$PGA_{\mathrm{BS\rightarrow M}} = 2V_{\mathrm{C\ BS}} + 1.5V_{\mathrm{O\ BS}} - PR_{\mathrm{BS}}. \quad 26$$

The MAL produced by MDH in the M diffuses to the BS, and the flux is:

$$MAL_{\mathrm{M\rightarrow BS}} = MDH_{\mathrm{M}}. \quad 27$$

Because no PEP is consumed in the BS and the sources of PEP in the BS are PEPCK and PPDK, the flux of PEP from BS to M is:

$$PEP_{\mathrm{BS\to M}} = PEPCK + PPDK_{\mathrm{BS}}. \quad 28$$

The ASP diffusing to the BS results from OAA transamination (Eqn 21) is:

$$ASP_{\mathrm{M\to BS}} = T. \quad 29$$

The fraction of GLY decarboxylated in the BS and not directly produced therein is imported from the M, the GLY flux from M to BS is calculated as:

$$GLY_{\mathrm{M\to BS}} = GDC_{\mathrm{BS}} - V_{\mathrm{O\,BS}}. \quad 30$$

GDC reacts GLY to ½SER and ½NH_3. SER diffuses to M as such, while the surplus NH_3 is rebalanced (*R*) so as to maintain a functional C_2 shuttle. It can therefore be written:

$$SER_{\mathrm{BS\to M}} = R = \frac{1}{2} GLY_{\mathrm{M\to BS}}\,. \quad 31$$

Glutamate and αKG do not diffuse directly between the M and BS, but exchange aminogroups with ALA and PYR (Mallmann *et al.*, 2014; Pick *et al.*, 2011). The flux of ALA from the BS to M corresponds to the fluxes of amino–groups imported in the BS through the CCM (i.e. transamination rate, *T*) plus the surplus flux of amino groups deriving from GLY decarboxylation:

$$ALA_{\mathrm{BS\to M}} = T + R\,. \quad 32$$

The flux of PYR from the BS to M balances the activity of ME (producing PYR in BS), *T* (consuming PYR in BS), the flux of PYR required to rebalance the amino–groups in the C_2 shuttle, and the activity of PPDK (consuming PYR in BS):

$$PYR_{\mathrm{BS\to M}} = ME - T + R - PPDK_{\mathrm{BS}}\,. \quad 33$$

Finally, the flux of CO_2 diffusing out of the BS, the leak rate (*L*) can be calculated by balancing all CO_2 fluxes in and out of the BS as:

$$L_{\mathrm{BS\to M}} = ME + PEPCK + R_{\mathrm{LIGHT\,BS}} + \frac{1}{2} GDC_{\mathrm{BS}} - V_{\mathrm{C\,BS}}\,. \quad 34$$

Several ratios are calculated in the Excel workbook, including leakiness ($\phi = L/V_P$) and the ATP cost of gross assimilation (*ATP*/*GA*), but these are self–explanatory (see Workbook, Fig. S1–S4E).

Simulation of static scenarios under physiological operational conditions

Table S1. Input quantities used to simulate photosynthetic types and example species in each group

**Type**	**C_3**	**C_2**		**C_2+C_4**				**C_4**		
Subtype	-	-	NADP–ME	NAD–ME	PCK	NADP–ME	NADP–ME (+PCK)	NAD–ME	PEPCK (NADP–ME)	PEPCK (NAD–ME)
Example	wheat	*Mollugo verticillata*	*Flaveria pubescens*	*Alternanthera tenella*	*Alloteropsis semialata*	sorghum	maize	*Panicum sp.* (sensu stricto)	*Alloteropsis semialata subsp. semialata*	*Spartina sp.*
Figure below	S2	S3	S4A	S4B	S4C	S5A	S5B	S5C	S5D	S5E
Simulation	1.1	2.1	3.1.1	3.1.2	3.1.3	4.1.1	4.1.2	4.1.3	4.1.4	4.1.5
**Basic quantities**										
R_{LIGHT} / μmol m^{-2} s^{-1}	**1**	**1**	**1**	**1**	**1**	**1**	**1**	**1**	**1**	**1**
A / μmol m^{-2} s^{-1}	**9**	**9**	**9**	**9**	**9**	**9**	**9**	**9**	**9**	**9**
$r_{O/C}$	**0.5**	**0.45**	**0.3**	**0.3**	**0.3**	**0.05**	**0.05**	**0.05**	**0.05**	**0.05**
**CCM**										
V_P / μmol m^{-2} s^{-1}	**0**	**0**	**3**	**3**	**3**	**10.85**	**10.85**	**10.85**	**10.85**	**10.85**
$PEPCK/V_P$	**0**	**0**	**0**	**0**	**1**	**0**	**0.2**	**0**	**1**	**1**
MDH_M	irrelevant	Eqn 16	Eqn 16	MDH_M=0	Eqn 16	Eqn 16	Eqn 16	MDH_M=0	Eqn 16	MDH_M=0
**BS contribution**										
**Slow response**										
f_C, f_O	**0**	**0.1**	**0.35**	**0.35**	**0.35**	**1**	**1**	**1**	**1**	**1**
f_{GDC}	**0**	**1**	**1**	**1**	**1**	**1**	**1**	**1**	**1**	**1**
f_{RLIGHT}	**0**	**0.1**	**0.3**	**0.3**	**0.3**	**0.5**	**0.5**	**0.5**	**0.5**	**0.5**
**Fast response**										
f_{PR}	**0**	**0.1**	**0.1**	**0.1**	**0.1**	**0.3**	**0.3**	**0.3**	**0.3**	**0.3**
f_{PPDK}	**0**	**0**	**0**	**0**	**0**	**0**	**0**	**0**	**0**	**0**
f_{CS}	**0**	**0**	**0**	**0**	**0**	**0**	**0**	**0**	**0**	**0**

Figures.

Figure S1. Simulation 1.1. Output for a C_3 photosynthetic type. Input quantities are shown in Table S1. Output rates are given next to the metabolite name or the flux arrow. Further output is shown in the Spreadsheet snapshot below. Units are consistent with *A* (µmol m^{-2} s^{-1}).

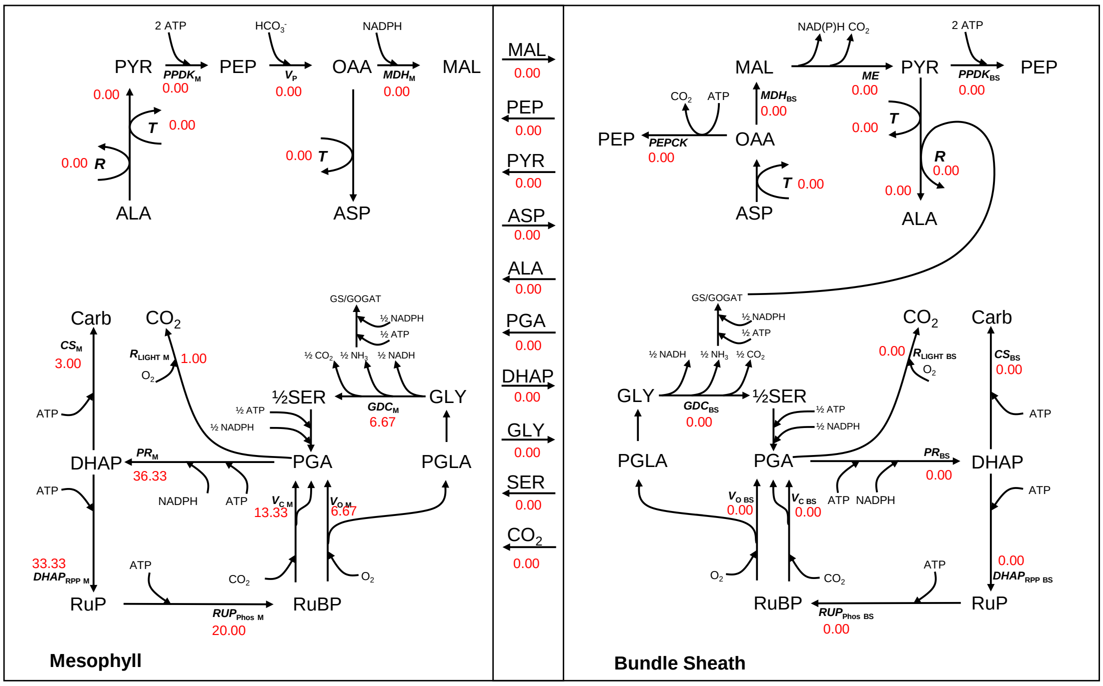

Input

Basic quantities	
R_{LIGHT}	1
A	9
V_O/V_C	0.5

CCM	
V_P	0.00
r_{PEPCK}	0.00

BS engagement	
**Slow response**	
f_C	0
f_O	0
f_{GDC}	0
f_{RLIGHT}	0
**Fast response**	
f_{PR}	0
f_{CS}	0
f_{PPDK}	0

Output

Overall rates	
GA	10.00
V_C	13.33
V_O	6.67
Carb synthesis CS_{TOT}	3.00
PGA reduction PR_{TOT}	36.33
DHAP entering RPP	33.33
RuP phosphorylation	20.00
GDC_{TOT}	6.67
NADPH demand Tot	39.67
ATP demand Tot	64.50

CCM	
V_P	0.00
$PEPCK_{MAX}$ (=V_P)	0.00
$PEPCK$	0.00
$PPDK_{TOT}$	0.00

BS/M partitioning	in BS	in M
V_C	0.00	13.33
V_O	0.00	6.67
GDC	0.00	6.67
R_{LIGHT}	0.00	1.00
Carb synthesis CS	0.00	3.00
$PPDK$	0.00	0.00
PGA reduction PR	0.00	36.33
DHAP entering RPP	0.00	33.33
RuP phosphorylation	0.00	20.00

Reducing power balance	in BS	in M
NADPH potent. avail. through CCM	0.00	-
NADPH demand (excl. CCM)	0.00	39.67
MDH	0.00	0.00
Transamination T	0.00	0.00
ME	0.00	-
NADPH demand (incl. CCM)	0.00	39.67
NADPH demand thr. LEF	0.00	39.67
ATP demand	0.00	64.50

Ratios	
ATP_{TOT}/GA	6.450
Leakiness ϕ	-
$ATP_{TOT}/NADPH_{TOT}$	1.626
$NADPH_{BS}/NADPH_{TOT}$	0.000
$NADPH_{BS}/NADPH_M$	0.000
ATP_{BS}/ATP_M	0.000
ATP_{BS}/ATP_{TOT}	0.000
Photo-prod. $ATP_{BS}/NADPH_{BS}$	-
Photo-prod. $ATP_M/NADPH_M$	1.626
ASP/MAL decarboxilation	-
T/V_P	-

Fluxes	
CO_2 BS→M Leak rate L	0.00
DHAP M→BS	0.00
PGA BS→M	0.00
MAL M→BS	0.00
PEP BS→M	0.00
ASP M→BS	0.00
GLY M→BS	0.00
SER BS→M =R	0.00
ALA BS→M	0.00
PYR BS→M	0.00
Total fluxes	0

Figure S2. Simulation 1.2. Energetics involved following the manipulation of photorespiration (as Rubisco rate of oxygenation versus carboxylation, $r_{O/C}$) in a C_3 photosynthetic type. Other inputs are shown in Table 3.

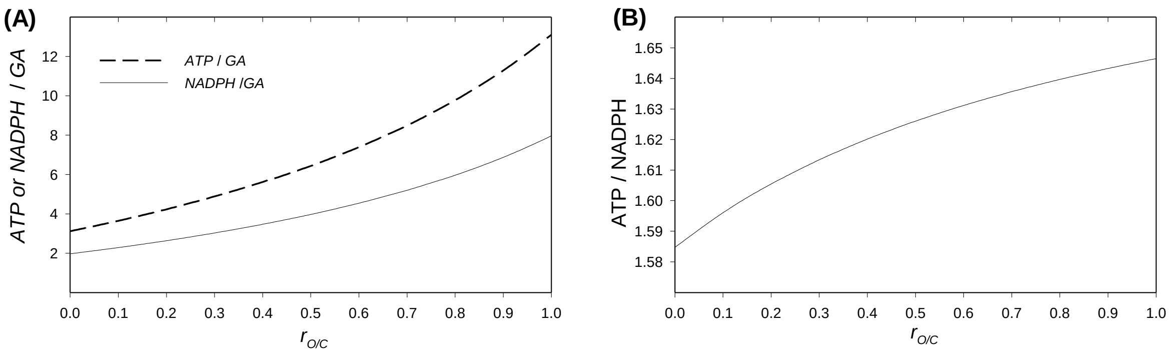

Figure S3. Simulation 2.1. SMA output for a typical C_2 photosynthetic type. Input quantities are shown in Table S1. Output rates are given next to the metabolite name or the flux arrow. Further output is shown in the Spreadsheet snapshot below. Negative fluxes have orientation opposite to that of the arrow. Units are consistent with *A* (μmol m^{-2} s^{-1}).

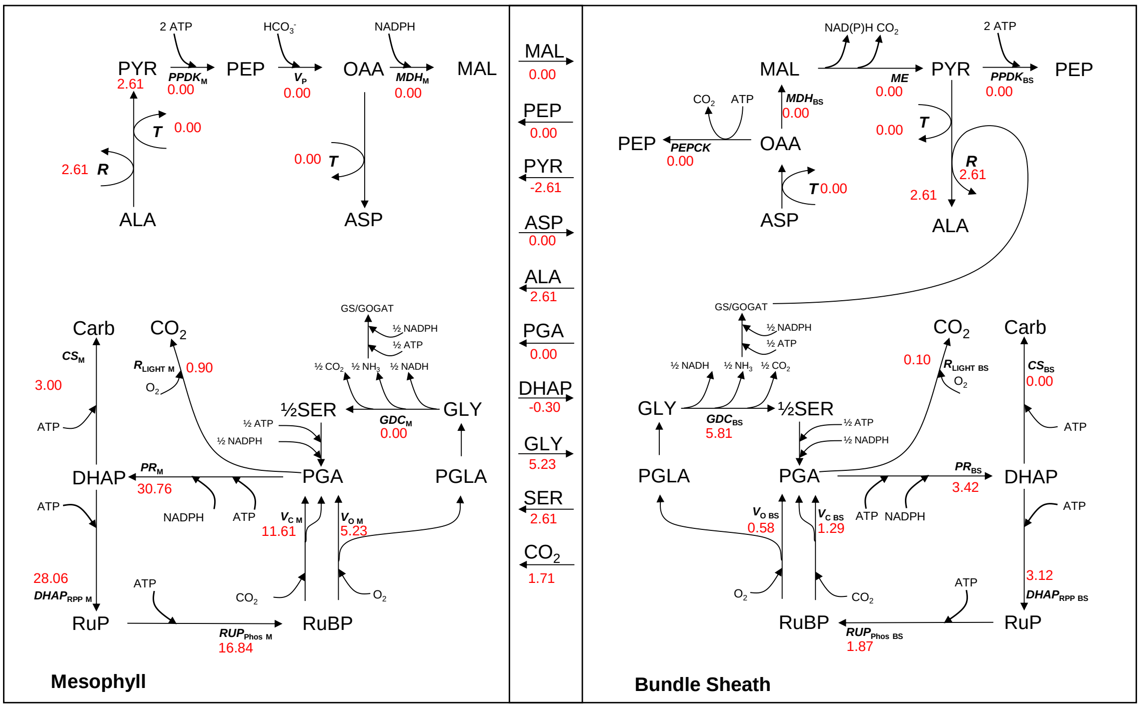

Input

Basic quantities	
R_{LIGHT}	1
A	9
V_O/V_C	0.45

CCM	
V_P	0.00
r_{PEPCK}	0.00

BS engagement	
Slow response	
f_C	0.1
f_O	0.1
f_{GDC}	1
f_{RLIGHT}	0.1
Fast response	
f_{PR}	0.1
f_{CS}	0
f_{PPDK}	0

Output

Overall rates	
GA	10.00
V_C	12.90
V_O	5.81
Carb synthesis CS_{TOT}	3.00
PGA reduction PR_{TOT}	34.18
DHAP entering RPP	31.18
RuP phosphorylation	18.71
GDC_{TOT}	5.81
NADPH demand Tot	37.09
ATP demand Tot	60.20

CCM	
V_P	0.00
$PEPCK_{MAX}$ (=V_P)	0.00
PEPCK	0.00
$PPDK_{TOT}$	0.00

BS/M partitioning	in BS	in M
V_C	1.29	11.61
V_O	0.58	5.23
GDC	5.81	0.00
R_{LIGHT}	0.10	0.90
Carb synthesis *CS*	0.00	3.00
PPDK	0.00	0.00
PGA reduction *PR*	3.42	30.76
DHAP entering RPP	3.12	28.06
RuP phosphorylation	1.87	16.84

Reducing power balance	in BS	in M
NADPH potent. avail. through CCM	0.00	-
NADPH demand (excl. CCM)	1.10	35.99
MDH	0.00	0.00
Transamination *T*	0.00	0.00
ME	0.00	-
NADPH demand (incl. CCM)	1.10	35.99
NADPH demand thr. LEF	1.10	35.99
ATP demand	8.48	51.72

Ratios	
ATP_{TOT}/GA	6.020
Leakiness ϕ	-
$ATP_{TOT}/NADPH_{TOT}$	1.623
$NADPH_{BS}/NADPH_{TOT}$	0.030
$NADPH_{BS}/NADPH_M$	0.030
ATP_{BS}/ATP_M	0.164
ATP_{BS}/ATP_{TOT}	0.141
Photo-prod. $ATP_{BS}/NADPH_{BS}$	7.742
Photo-prod. $ATP_M/NADPH_M$	1.437
ASP/MAL decarboxilation	-
T/V_P	-

Fluxes	
CO_2 BS→M Leak rate *L*	1.71
DHAP M→BS	-0.30
PGA BS→M	0.00
MAL M→BS	0.00
PEP BS→M	0.00
ASP M→BS	0.00
GLY M→BS	5.23
SER BS→M =*R*	2.61
ALA BS→M	2.61
PYR BS→M	-2.61
Total fluxes	15

Figure S4A. Simulation 3.1.1. SMA output for a NADP–ME C_2+C_4 photosynthetic subtype. Input quantities are shown in Table S1. Output rates are given next to the metabolite name or the flux arrow. Further output is shown in the Spreadsheet snapshot below. Units are consistent with *A* (μmol m^{-2} s^{-1}).

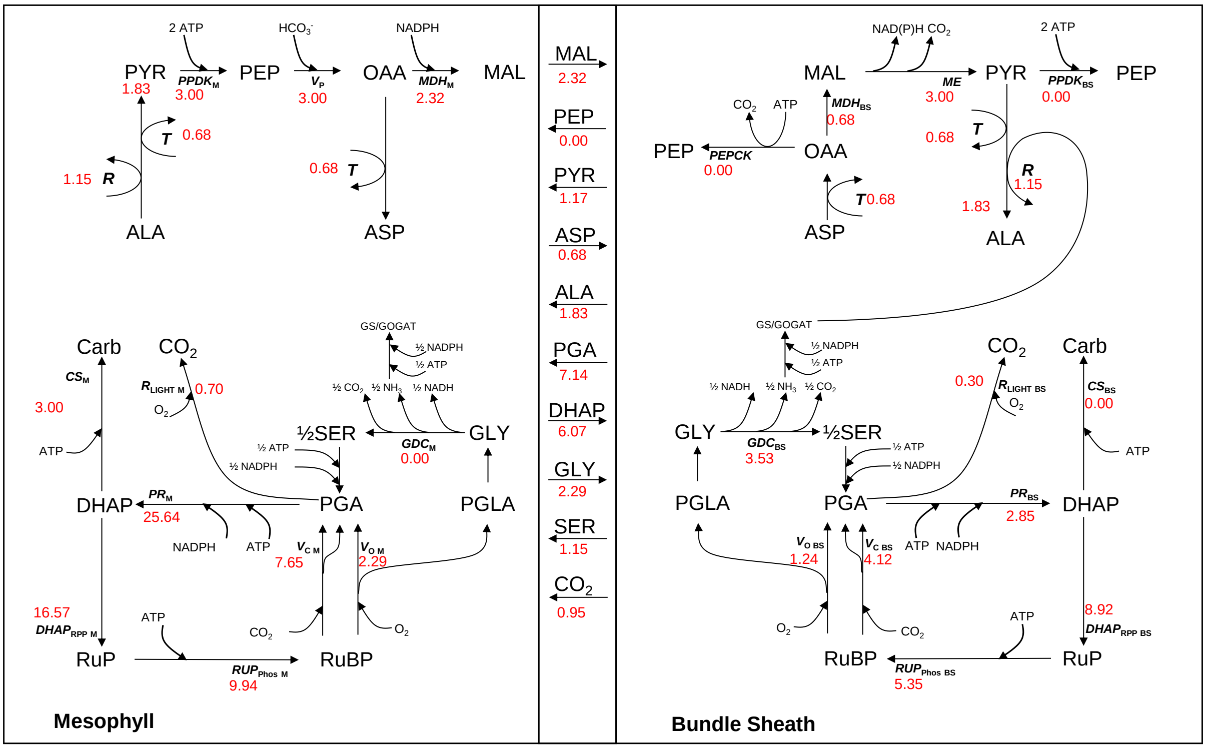

Input

Basic quantities	
R_{LIGHT}	1
A	9
V_O/V_C	0.3

CCM	
V_P	3.00
r_{PEPCK}	0.00

BS engagement	
**Slow response**	
f_C	0.35
f_O	0.35
f_{GDC}	1
f_{RLIGHT}	0.3
**Fast response**	
f_{PR}	0.1
f_{CS}	0
f_{PPDK}	0

Output

Overall rates	
GA	10.00
V_C	11.76
V_O	3.53
Carb synthesis CS_{TOT}	3.00
PGA reduction PR_{TOT}	28.49
DHAP entering RPP	25.49
RuP phosphorylation	15.29
GDC_{TOT}	3.53
NADPH demand Tot	30.25
ATP demand Tot	54.81

CCM	
V_P	3.00
$PEPCK_{MAX}$ (=V_P)	3.00
PEPCK	0.00
$PPDK_{TOT}$	3.00

BS/M partitioning	in BS	in M
V_C	4.12	7.65
V_O	1.24	2.29
GDC	3.53	0.00
R_{LIGHT}	0.30	0.70
Carb synthesis *CS*	0.00	3.00
PPDK	0.00	3.00
PGA reduction *PR*	2.85	25.64
DHAP entering RPP	8.92	16.57
RuP phosphorylation	5.35	9.94

Reducing power balance	in BS	in M
NADPH potent. avail. through CCM	3.00	-
NADPH demand (excl. CCM)	2.32	27.94
MDH	0.68	2.32
Transamination *T*	0.68	0.68
ME	3.00	-
NADPH demand (incl. CCM)	0.00	30.25
NADPH demand thr. LEF	0.00	30.25
ATP demand	10.58	44.23

Ratios	
ATP_{TOT}/GA	5.481
Leakiness ϕ	0.32
$ATP_{TOT}/NADPH_{TOT}$	1.812
$NADPH_{BS}/NADPH_{TOT}$	0.077
$NADPH_{BS}/NADPH_M$	0.083
ATP_{BS}/ATP_M	0.239
ATP_{BS}/ATP_{TOT}	0.193
Photo-prod. $ATP_{BS}/NADPH_{BS}$	CEF only
Photo-prod. $ATP_M/NADPH_M$	1.462
ASP/MAL decarboxilation	0.293
T/V_P	0.227

Fluxes	
CO_2 BS→M Leak rate *L*	0.95
DHAP M→BS	6.07
PGA BS→M	7.14
MAL M→BS	2.32
PEP BS→M	0.00
ASP M→BS	0.68
GLY M→BS	2.29
SER BS→M =*R*	1.15
ALA BS→M	1.83
PYR BS→M	1.17
Total fluxes	24

Figure S4B. Simulation 3.1.2. SMA output for a NAD–ME C_2+C_4 photosynthetic subtype. Input quantities are shown in Table S1. Output rates are given next to the metabolite name or the flux arrow. Further output is shown in the Spreadsheet snapshot below. Units are consistent with *A* (μmol m^{-2} s^{-1}).

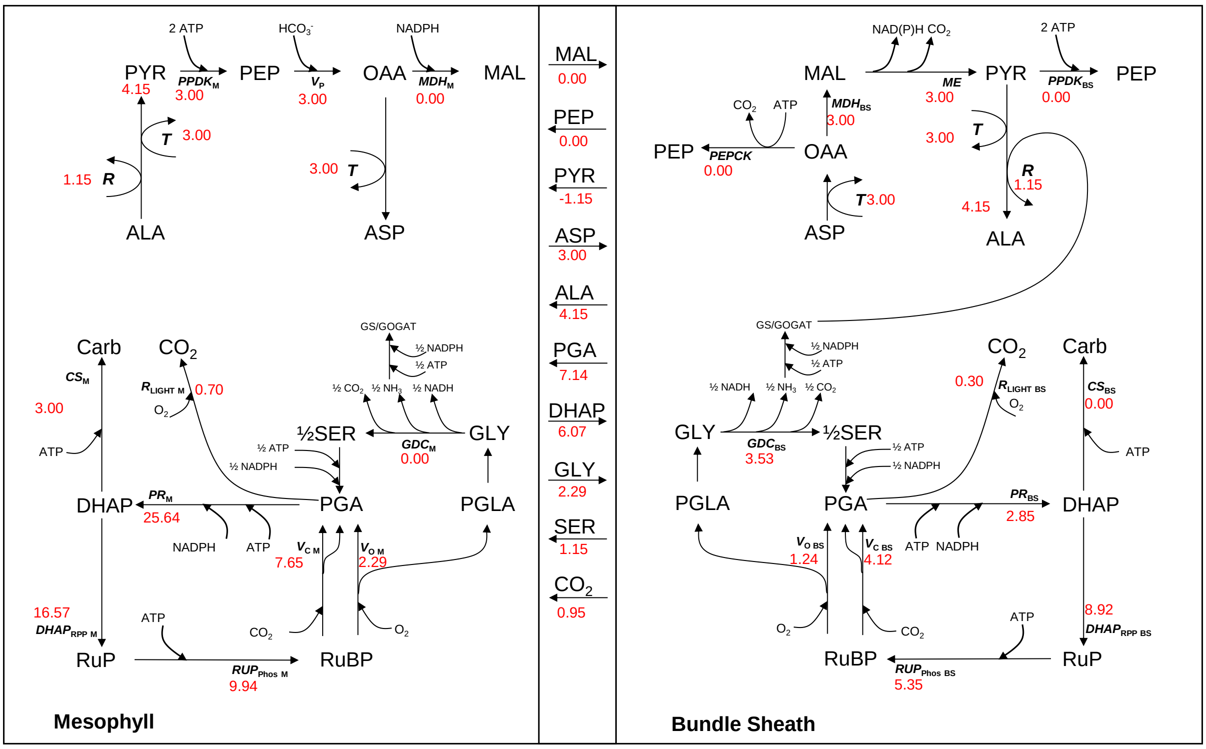

Input

Basic quantities	
R_{LIGHT}	1
A	9
V_O/V_C	0.3

CCM	
V_P	3.00
r_{PEPCK}	0.00

BS engagement	
**Slow response**	
f_C	0.35
f_O	0.35
f_{GDC}	1
f_{RLIGHT}	0.3
**Fast response**	
f_{PR}	0.1
f_{CS}	0
f_{PPDK}	0

Output

Overall rates	
GA	10.00
V_C	11.76
V_O	3.53
Carb synthesis CS_{TOT}	3.00
PGA reduction PR_{TOT}	28.49
DHAP entering RPP	25.49
RuP phosphorylation	15.29
GDC_{TOT}	3.53
NADPH demand Tot	30.25
ATP demand Tot	54.81

CCM	
V_P	3.00
$PEPCK_{MAX}$ (=V_P)	3.00
PEPCK	0.00
$PPDK_{TOT}$	3.00

BS/M partitioning	in BS	in M
V_C	4.12	7.65
V_O	1.24	2.29
GDC	3.53	0.00
R_{LIGHT}	0.30	0.70
Carb synthesis *CS*	0.00	3.00
PPDK	0.00	3.00
PGA reduction *PR*	2.85	25.64
DHAP entering RPP	8.92	16.57
RuP phosphorylation	5.35	9.94

Reducing power balance	in BS	in M
NADPH potent. avail. through CCM	3.00	-
NADPH demand (excl. CCM)	2.32	27.94
MDH	3.00	0.00
Transamination *T*	3.00	3.00
ME	3.00	-
NADPH demand (incl. CCM)	2.32	27.94
NADPH demand thr. LEF	2.32	27.94
ATP demand	10.58	44.23

Ratios	
ATP_{TOT}/GA	5.481
Leakiness ϕ	0.32
$ATP_{TOT}/NADPH_{TOT}$	1.812
$NADPH_{BS}/NADPH_{TOT}$	0.077
$NADPH_{BS}/NADPH_M$	0.083
ATP_{BS}/ATP_M	0.239
ATP_{BS}/ATP_{TOT}	0.193
Photo-prod. $ATP_{BS}/NADPH_{BS}$	4.563
Photo-prod. $ATP_M/NADPH_M$	1.583
ASP/MAL decarboxilation	-
T/V_P	1.000

Fluxes	
CO_2 BS→M Leak rate *L*	0.95
DHAP M→BS	6.07
PGA BS→M	7.14
MAL M→BS	0.00
PEP BS→M	0.00
ASP M→BS	3.00
GLY M→BS	2.29
SER BS→M =*R*	1.15
ALA BS→M	4.15
PYR BS→M	-1.15
Total fluxes	26

Figure S4C. Simulation 3.1.3.SMA output for a PEPCK C_2+C_4 photosynthetic subtype. Input quantities are shown in Table S1. Output rates are given next to the metabolite name or the flux arrow. Further output is shown in the Spreadsheet snapshot below. Units are consistent with A (μmol m^{-2} s^{-1}).

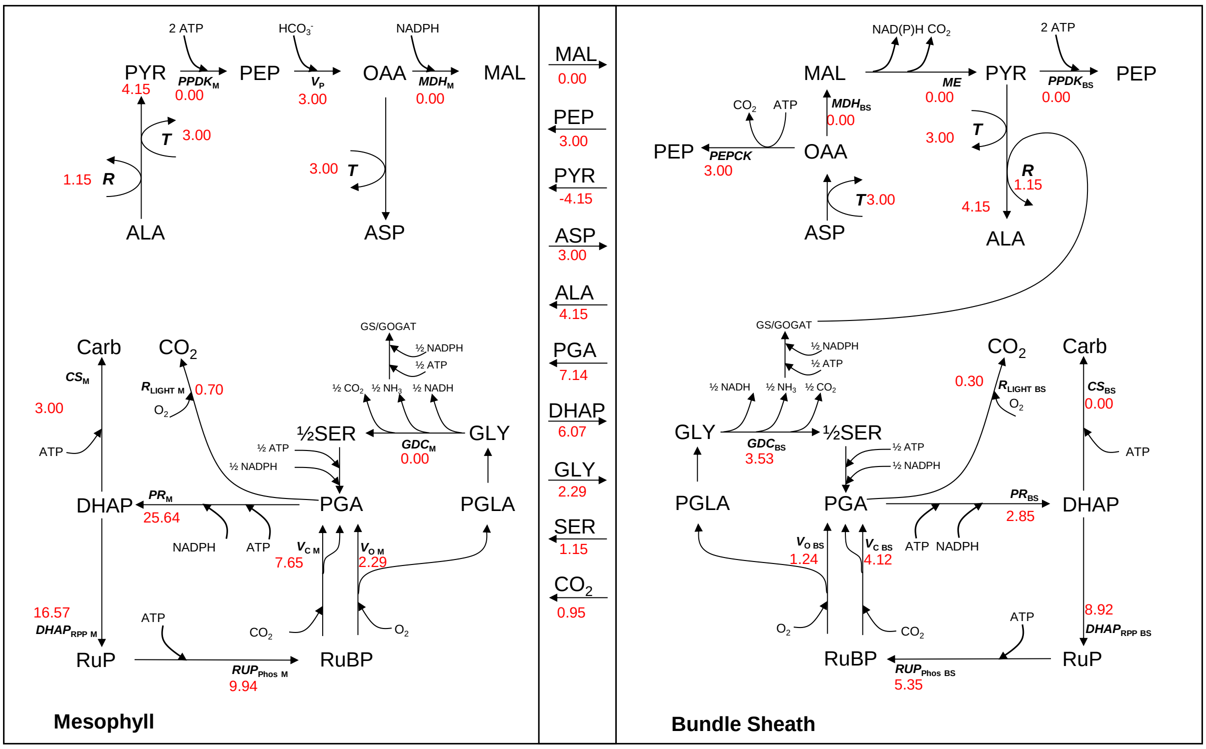

Input

Basic quantities	
R_{LIGHT}	1
A	9
V_O/V_C	0.3

CCM	
V_P	3.00
r_{PEPCK}	1.00

BS engagement	
Slow response	
f_C	0.35
f_O	0.35
f_{GDC}	1
f_{RLIGHT}	0.3
Fast response	
f_{PR}	0.1
f_{CS}	0
f_{PPDK}	0

Output

Overall rates	
GA	10.00
V_C	11.76
V_O	3.53
Carb synthesis CS_{TOT}	3.00
PGA reduction PR_{TOT}	28.49
DHAP entering RPP	25.49
RuP phosphorylation	15.29
GDC_{TOT}	3.53
NADPH demand Tot	30.25
ATP demand Tot	51.81

CCM	
V_P	3.00
$PEPCK_{MAX}$ (=V_P)	3.00
$PEPCK$	3.00
$PPDK_{TOT}$	0.00

BS/M partitioning	in BS	in M
V_C	4.12	7.65
V_O	1.24	2.29
GDC	3.53	0.00
R_{LIGHT}	0.30	0.70
Carb synthesis CS	0.00	3.00
$PPDK$	0.00	0.00
PGA reduction PR	2.85	25.64
DHAP entering RPP	8.92	16.57
RuP phosphorylation	5.35	9.94

Reducing power balance	in BS	in M
NADPH potent. avail. through CCM	0.00	-
NADPH demand (excl. CCM)	2.32	27.94
MDH	0.00	0.00
Transamination T	3.00	3.00
ME	0.00	-
NADPH demand (incl. CCM)	2.32	27.94
NADPH demand thr. LEF	2.32	27.94
ATP demand	13.58	38.23

Ratios	
ATP_{TOT}/GA	5.181
Leakiness ϕ	0.32
$ATP_{TOT}/NADPH_{TOT}$	1.713
$NADPH_{BS}/NADPH_{TOT}$	0.077
$NADPH_{BS}/NADPH_M$	0.083
ATP_{BS}/ATP_M	0.355
ATP_{BS}/ATP_{TOT}	0.262
Photo-prod. $ATP_{BS}/NADPH_{BS}$	5.856
Photo-prod. $ATP_M/NADPH_M$	1.368
ASP/MAL decarboxilation	-
T/V_P	1.000

Fluxes	
CO_2 BS→M Leak rate L	0.95
DHAP M→BS	6.07
PGA BS→M	7.14
MAL M→BS	0.00
PEP BS→M	3.00
ASP M→BS	3.00
GLY M→BS	2.29
SER BS→M =R	1.15
ALA BS→M	4.15
PYR BS→M	-4.15
Total fluxes	32

Figure S5A. Simulation 4.1.1. SMA output for a typical NADP–ME C_4 photosynthetic subtype. Input quantities are shown in Table S1. Output rates are given next to the metabolite name or the flux arrow. Further output is shown in the Spreadsheet snapshot below. Units are consistent with *A* (μmol m^{-2} s^{-1}).

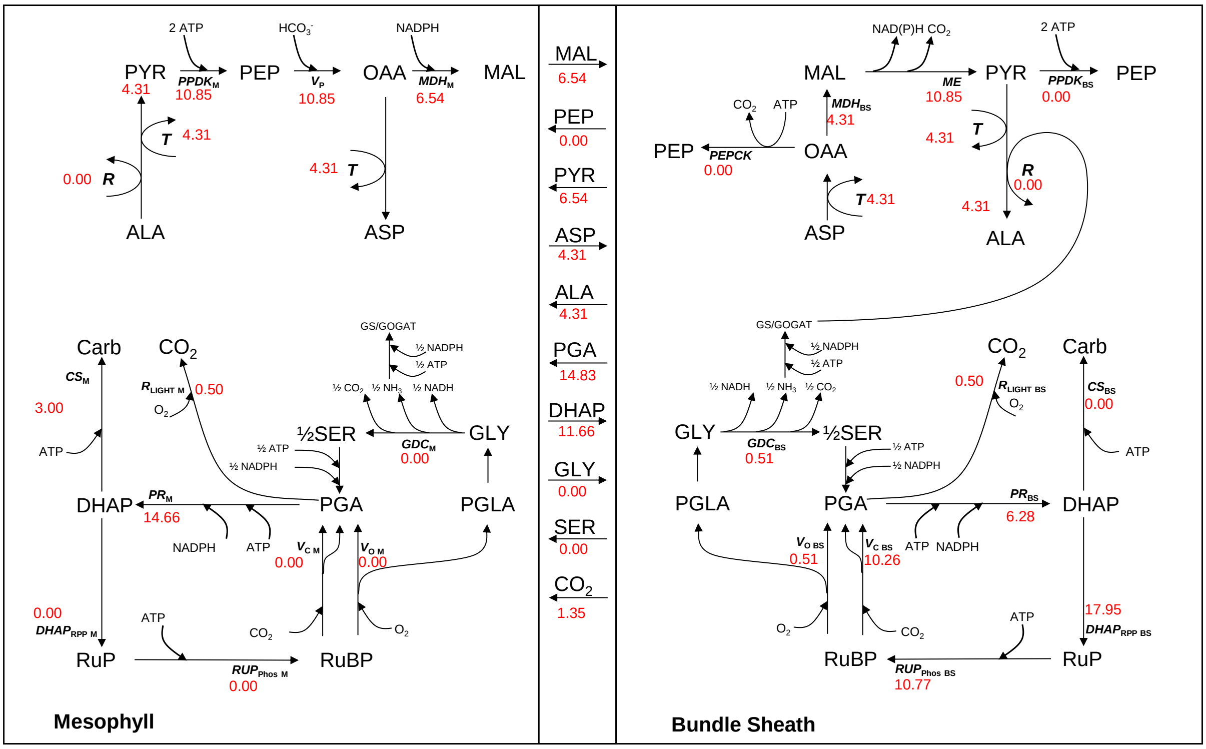

**Input**	
**Basic quantities**	
R_{LIGHT}	1
A	9
V_O/V_C	0.05
**CCM**	
V_P	10.85
r_{PEPCK}	0.00
**BS engagement**	
**Slow response**	
f_C	1
f_O	1
f_{GDC}	1
f_{RLIGHT}	0.5
**Fast response**	
f_{PR}	0.3
f_{CS}	0
f_{PPDK}	0

Output

**Overall rates**	
GA	10.00
V_C	10.26
V_O	0.51
Carb synthesis CS_{TOT}	3.00
PGA reduction PR_{TOT}	20.95
DHAP entering RPP	17.95
RuP phosphorylation	10.77
GDC_{TOT}	0.51
NADPH demand Tot	21.21
ATP demand Tot	55.43

**CCM**	
V_P	10.85
$PEPCK_{MAX}$ (=V_P)	10.85
PEPCK	0.00
$PPDK_{TOT}$	10.85

**BS/M partitioning**	**in BS**	**in M**
V_C	10.26	0.00
V_O	0.51	0.00
GDC	0.51	0.00
R_{LIGHT}	0.50	0.50
Carb synthesis *CS*	0.00	3.00
PPDK	0.00	10.85
PGA reduction *PR*	6.28	14.66
DHAP entering RPP	17.95	0.00
RuP phosphorylation	10.77	0.00

**Reducing power balance**	**in BS**	**in M**
NADPH potent. avail. through CCM	10.85	-
NADPH demand (excl. CCM)	6.54	14.66
MDH	4.31	6.54
Transamination *T*	4.31	4.31
ME	10.85	-
NADPH demand (incl. CCM)	0.00	21.21
NADPH demand thr. LEF	0.00	21.21
ATP demand	17.57	37.86

**Ratios**	
ATP_{TOT}/GA	5.543
Leakiness ϕ	0.12
$ATP_{TOT}/NADPH_{TOT}$	2.614
$NADPH_{BS}/NADPH_{TOT}$	0.308
$NADPH_{BS}/NADPH_M$	0.446
ATP_{BS}/ATP_M	0.464
ATP_{BS}/ATP_{TOT}	0.317
Photo-prod. $ATP_{BS}/NADPH_{BS}$	CEF only
Photo-prod. $ATP_M/NADPH_M$	1.786
ASP/MAL decarboxilation	0.659
T/V_P	0.397

**Fluxes**	
CO_2 BS→M Leak rate *L*	1.35
DHAP M→BS	11.66
PGA BS→M	14.83
MAL M→BS	6.54
PEP BS→M	0.00
ASP M→BS	4.31
GLY M→BS	0.00
SER BS→M =*R*	0.00
ALA BS→M	4.31
PYR BS→M	6.54
Total fluxes	50

Figure S5B. Simulation 4.1.2. SMA output for a typical NADP–ME C_4 photosynthetic subtype with engagement of PEPCK in the BS. Input quantities are shown in Table S1. Output rates are given next to the metabolite name or the flux arrow. Further output is shown in the Spreadsheet snapshot below. Units are consistent with A (μmol m^{-2} s^{-1}).

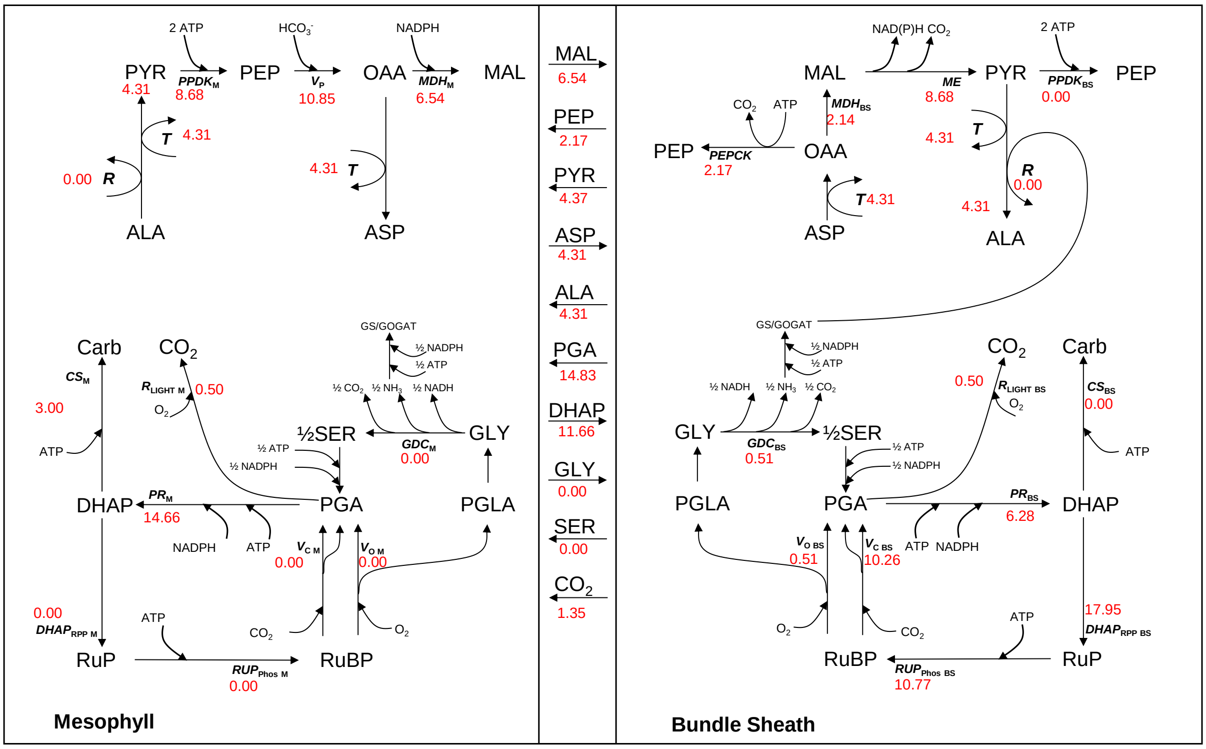

Input

Basic quantities	
R_{LIGHT}	1
A	9
V_O/V_C	0.05

CCM	
V_P	10.85
r_{PEPCK}	0.20

BS engagement	
**Slow response**	
f_C	1
f_O	1
f_{GDC}	1
f_{RLIGHT}	0.5
**Fast response**	
f_{PR}	0.3
f_{CS}	0
f_{PPDK}	0

Output

Overall rates	
GA	10.00
V_C	10.26
V_O	0.51
Carb synthesis CS_{TOT}	3.00
PGA reduction PR_{TOT}	20.95
DHAP entering RPP	17.95
RuP phosphorylation	10.77
GDC_{TOT}	0.51
NADPH demand Tot	21.21
ATP demand Tot	53.26

CCM	
V_P	10.85
$PEPCK_{MAX}$ (=V_P)	10.85
PEPCK	2.17
$PPDK_{TOT}$	8.68

BS/M partitioning	in BS	in M
V_C	10.26	0.00
V_O	0.51	0.00
GDC	0.51	0.00
R_{LIGHT}	0.50	0.50
Carb synthesis *CS*	0.00	3.00
PPDK	0.00	8.68
PGA reduction *PR*	6.28	14.66
DHAP entering RPP	17.95	0.00
RuP phosphorylation	10.77	0.00

Reducing power balance	in BS	in M
NADPH potent. avail. through CCM	8.68	-
NADPH demand (excl. CCM)	6.54	14.66
MDH	2.14	6.54
Transamination *T*	4.31	4.31
ME	8.68	-
NADPH demand (incl. CCM)	0.00	21.21
NADPH demand thr. LEF	0.00	21.21
ATP demand	19.74	33.52

Ratios	
ATP_{TOT}/GA	5.326
Leakiness ϕ	0.12
$ATP_{TOT}/NADPH_{TOT}$	2.512
$NADPH_{BS}/NADPH_{TOT}$	0.308
$NADPH_{BS}/NADPH_M$	0.446
ATP_{BS}/ATP_M	0.589
ATP_{BS}/ATP_{TOT}	0.371
Photo-prod. $ATP_{BS}/NADPH_{BS}$	CEF only
Photo-prod. $ATP_M/NADPH_M$	1.581
ASP/MAL decarboxilation	0.659
T/V_P	0.397

Fluxes	
CO_2 BS→M Leak rate *L*	1.35
DHAP M→BS	11.66
PGA BS→M	14.83
MAL M→BS	6.54
PEP BS→M	2.17
ASP M→BS	4.31
GLY M→BS	0.00
SER BS→M =*R*	0.00
ALA BS→M	4.31
PYR BS→M	4.37
Total fluxes	50

Figure S5C. Simulation 4.1.3. SMA output for a typical NAD–ME C_4 photosynthetic subtype with no engagement of PEPCK in the BS. Input quantities are shown in Table S1. Output rates are given next to the metabolite name or the flux arrow. Further output is shown in the Spreadsheet snapshot below. Units are consistent with A (µmol m^{-2} s^{-1}).

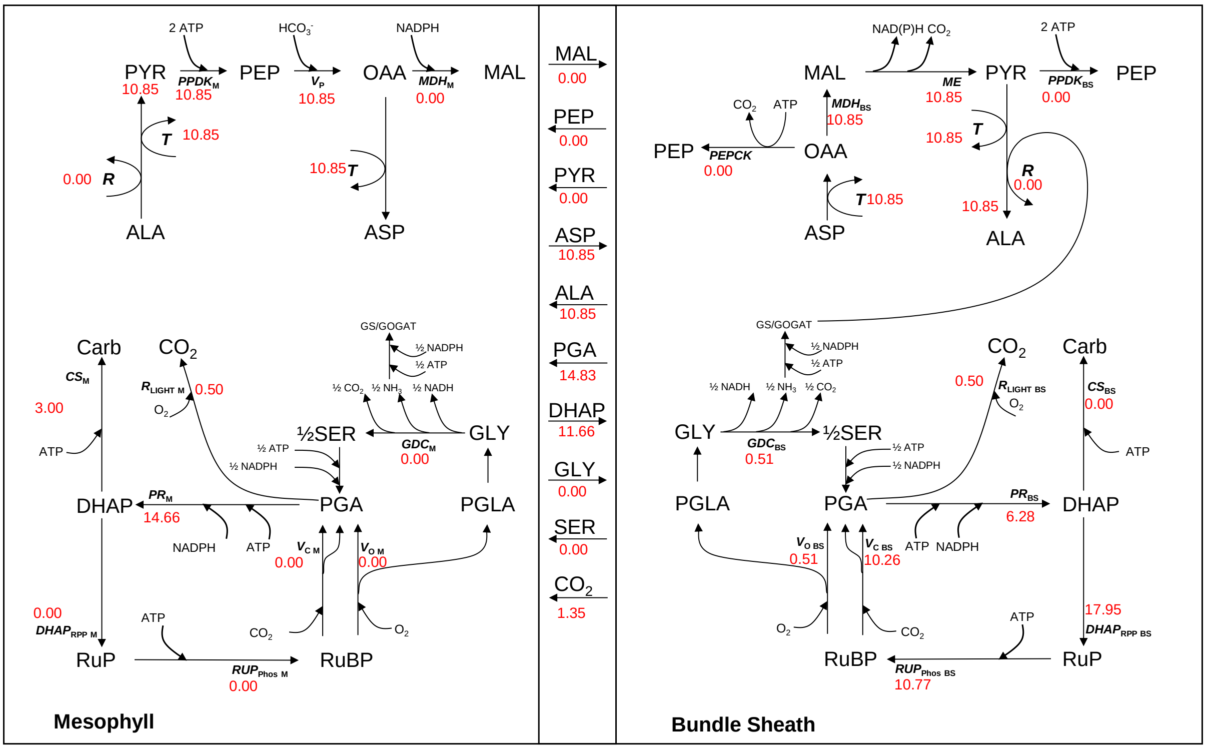

Input	
**Basic quantities**	
R_{LIGHT}	1
A	9
V_O/V_C	0.05
**CCM**	
V_P	10.85
r_{PEPCK}	0.00
**BS engagement**	
**Slow response**	
f_C	1
f_O	1
f_{GDC}	1
f_{RLIGHT}	0.5
**Fast response**	
f_{PR}	0.3
f_{CS}	0
f_{PPDK}	0

Output

Overall rates	
GA	10.00
V_C	10.26
V_O	0.51
Carb synthesis CS_{TOT}	3.00
PGA reduction PR_{TOT}	20.95
DHAP entering RPP	17.95
RuP phosphorylation	10.77
GDC_{TOT}	0.51
NADPH demand Tot	21.21
ATP demand Tot	55.43

CCM	
V_P	10.85
$PEPCK_{MAX}$ (=V_P)	10.85
$PEPCK$	0.00
$PPDK_{TOT}$	10.85

BS/M partitioning	in BS	in M
V_C	10.26	0.00
V_O	0.51	0.00
GDC	0.51	0.00
R_{LIGHT}	0.50	0.50
Carb synthesis CS	0.00	3.00
$PPDK$	0.00	10.85
PGA reduction PR	6.28	14.66
DHAP entering RPP	17.95	0.00
RuP phosphorylation	10.77	0.00

Reducing power balance	in BS	in M
NADPH potent. avail. through CCM	10.85	-
NADPH demand (excl. CCM)	6.54	14.66
MDH	10.85	0.00
Transamination T	10.85	10.85
ME	10.85	-
NADPH demand (incl. CCM)	6.54	14.66
NADPH demand thr. LEF	6.54	14.66
ATP demand	17.57	37.86

Ratios	
ATP_{TOT}/GA	5.543
Leakiness ϕ	0.12
$ATP_{TOT}/NADPH_{TOT}$	2.614
$NADPH_{BS}/NADPH_{TOT}$	0.308
$NADPH_{BS}/NADPH_M$	0.446
ATP_{BS}/ATP_M	0.464
ATP_{BS}/ATP_{TOT}	0.317
Photo-prod. $ATP_{BS}/NADPH_{BS}$	2.686
Photo-prod. $ATP_M/NADPH_M$	2.582
ASP/MAL decarboxilation	-
T/V_P	1.000

Fluxes	
CO_2 BS→M Leak rate L	1.35
DHAP M→BS	11.66
PGA BS→M	14.83
MAL M→BS	0.00
PEP BS→M	0.00
ASP M→BS	10.85
GLY M→BS	0.00
SER BS→M =R	0.00
ALA BS→M	10.85
PYR BS→M	0.00
Total fluxes	50

Figure S5D. Simulation 4.1.4. SMA output for a PEPCK (NADP–ME) C_4 photosynthetic subtype. Input quantities are shown in Table S1. Output rates are given next to the metabolite name or the flux arrow. Further output is shown in the Spreadsheet snapshot below. Units are consistent with A (μmol m^{-2} s^{-1}).

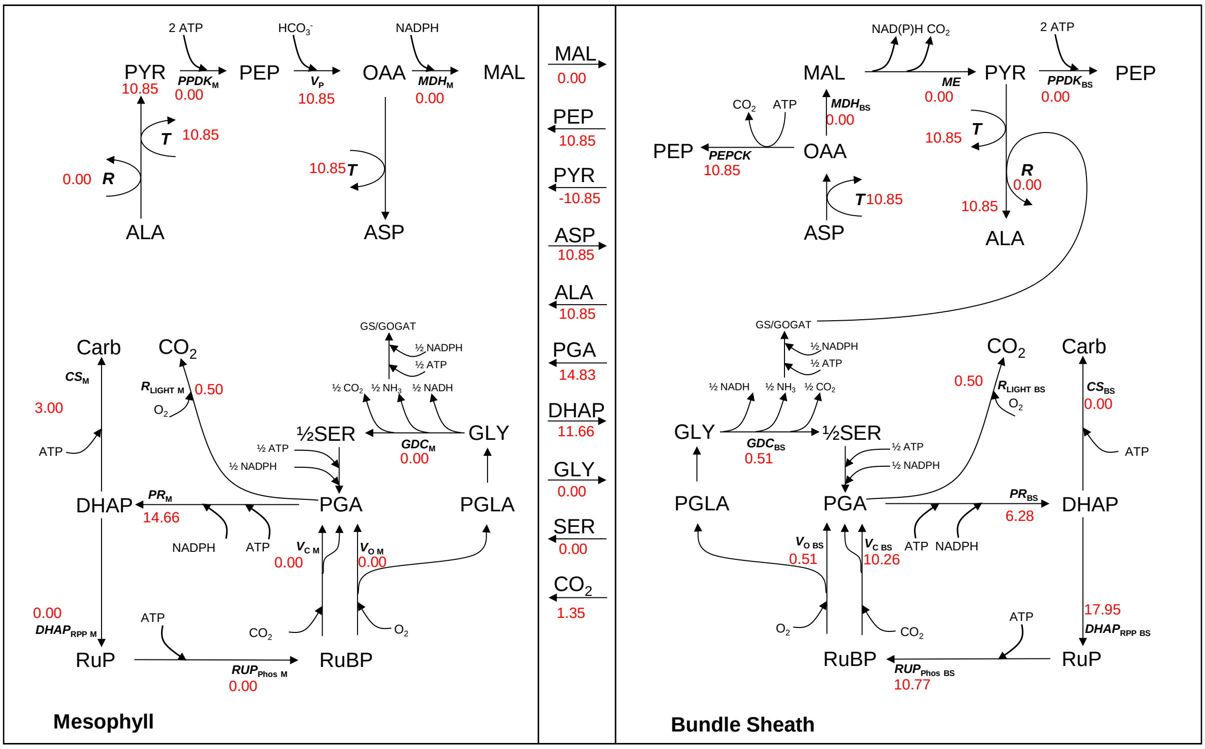

Input

Basic quantities	
R_{LIGHT}	1
A	9
V_O/V_C	0.05

CCM	
V_P	10.85
r_{PEPCK}	1.00

BS engagement	
Slow response	
f_C	1
f_O	1
f_{GDC}	1
f_{RLIGHT}	0.5
Fast response	
f_{PR}	0.3
f_{CS}	0
f_{PPDK}	0

Output

Overall rates	
GA	10.00
V_C	10.26
V_O	0.51
Carb synthesis CS_{TOT}	3.00
PGA reduction PR_{TOT}	20.95
DHAP entering RPP	17.95
RuP phosphorylation	10.77
GDC_{TOT}	0.51
NADPH demand Tot	21.21
ATP demand Tot	44.58

BS/M partitioning	in BS	in M
V_C	10.26	0.00
V_O	0.51	0.00
GDC	0.51	0.00
R_{LIGHT}	0.50	0.50
Carb synthesis CS	0.00	3.00
$PPDK$	0.00	0.00
PGA reduction PR	6.28	14.66
DHAP entering RPP	17.95	0.00
RuP phosphorylation	10.77	0.00

Ratios	
ATP_{TOT}/GA	4.458
Leakiness ϕ	0.12
$ATP_{TOT}/NADPH_{TOT}$	2.102
$NADPH_{BS}/NADPH_{TOT}$	0.308
$NADPH_{BS}/NADPH_{M}$	0.446
ATP_{BS}/ATP_{M}	1.758
ATP_{BS}/ATP_{TOT}	0.637
Photo-prod. $ATP_{BS}/NADPH_{BS}$	4.344
Photo-prod. $ATP_{M}/NADPH_{M}$	1.102
ASP/MAL decarboxilation	-
T/V_P	1.000

CCM	
V_P	10.85
$PEPCK_{MAX}$ (=V_P)	10.85
$PEPCK$	10.85
$PPDK_{TOT}$	0.00

Reducing power balance	in BS	in M
NADPH potent. avail. through CCM	0.00	-
NADPH demand (excl. CCM)	6.54	14.66
MDH	0.00	0.00
Transamination T	10.85	10.85
ME	0.00	-
NADPH demand (incl. CCM)	6.54	14.66
NADPH demand thr. LEF	6.54	14.66
ATP demand	28.42	16.16

Fluxes	
CO_2 BS→M Leak rate L	1.35
DHAP M→BS	11.66
PGA BS→M	14.83
MAL M→BS	0.00
PEP BS→M	10.85
ASP M→BS	10.85
GLY M→BS	0.00
SER BS→M =R	0.00
ALA BS→M	10.85
PYR BS→M	-10.85
Total fluxes	71

Figure S5E. Simulation 4.1.5. SMA output for a PEPCK (NAD–ME) C_4 photosynthetic subtype. Input quantities are shown in Table S1. Output rates are given next to the metabolite name or the flux arrow. Further output is shown in the Spreadsheet snapshot below. Units are consistent with *A* (μmol m^{-2} s^{-1}).

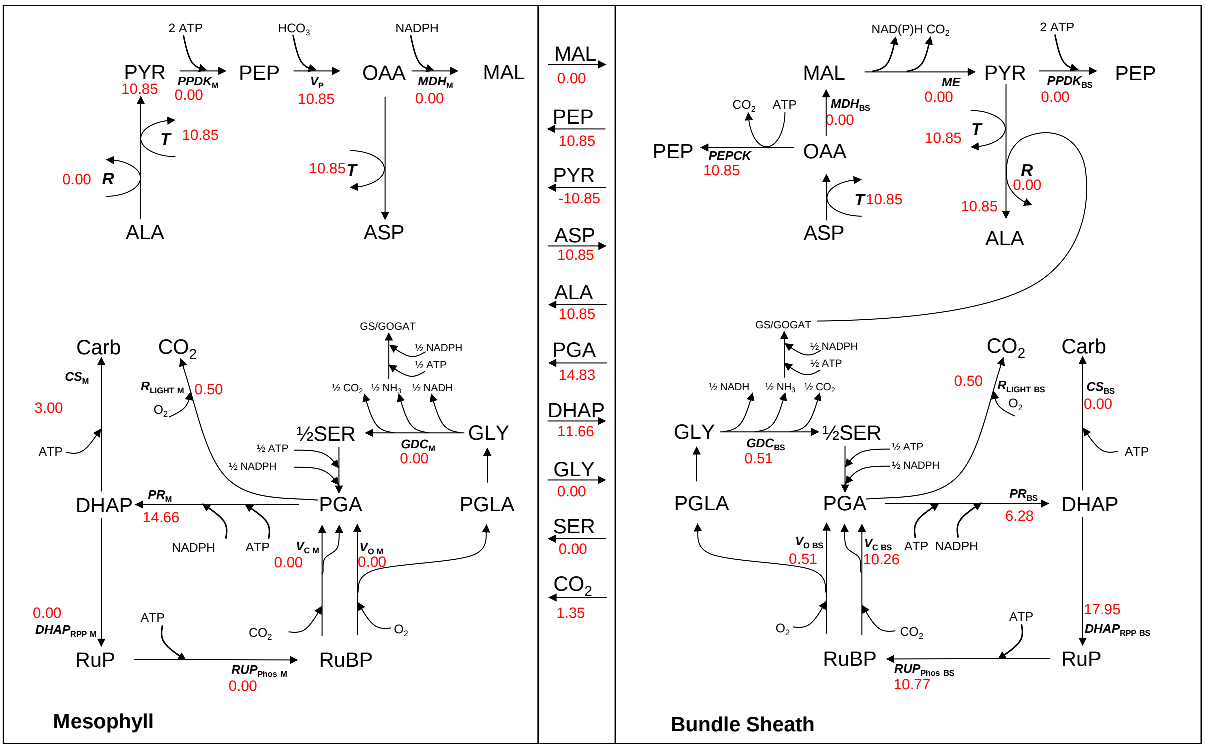

Input	
**Basic quantities**	
R_{LIGHT}	1
A	9
V_O/V_C	0.05
**CCM**	
V_P	10.85
r_{PEPCK}	1.00
**BS engagement**	
**Slow response**	
f_C	1
f_O	1
f_{GDC}	1
f_{RLIGHT}	0.5
**Fast response**	
f_{PR}	0.3
f_{CS}	0
f_{PPDK}	0

Output

Overall rates	
GA	10.00
V_C	10.26
V_O	0.51
Carb synthesis CS_{TOT}	3.00
PGA reduction PR_{TOT}	20.95
DHAP entering RPP	17.95
RuP phosphorylation	10.77
GDC_{TOT}	0.51
NADPH demand Tot	21.21
ATP demand Tot	44.58

CCM	
V_P	10.85
$PEPCK_{MAX}$ (=V_P)	10.85
PEPCK	10.85
$PPDK_{TOT}$	0.00

BS/M partitioning	in BS	in M
V_C	10.26	0.00
V_O	0.51	0.00
GDC	0.51	0.00
R_{LIGHT}	0.50	0.50
Carb synthesis *CS*	0.00	3.00
PPDK	0.00	0.00
PGA reduction *PR*	6.28	14.66
DHAP entering RPP	17.95	0.00
RuP phosphorylation	10.77	0.00

Reducing power balance	in BS	in M
NADPH potent. avail. through CCM	0.00	-
NADPH demand (excl. CCM)	6.54	14.66
MDH	0.00	0.00
Transamination *T*	10.85	10.85
ME	0.00	-
NADPH demand (incl. CCM)	6.54	14.66
NADPH demand thr. LEF	6.54	14.66
ATP demand	28.42	16.16

Ratios	
ATP_{TOT}/GA	4.458
Leakiness ϕ	0.12
$ATP_{TOT}/NADPH_{TOT}$	2.102
$NADPH_{BS}/NADPH_{TOT}$	0.308
$NADPH_{BS}/NADPH_{M}$	0.446
ATP_{BS}/ATP_{M}	1.758
ATP_{BS}/ATP_{TOT}	0.637
Photo-prod. $ATP_{BS}/NADPH_{BS}$	4.344
Photo-prod. $ATP_{M}/NADPH_{M}$	1.102
ASP/MAL decarboxilation	-
T/V_P	1.000

Fluxes	
CO_2 BS→M Leak rate *L*	1.35
DHAP M→BS	11.66
PGA BS→M	14.83
MAL M→BS	0.00
PEP BS→M	10.85
ASP M→BS	10.85
GLY M→BS	0.00
SER BS→M =*R*	0.00
ALA BS→M	10.85
PYR BS→M	-10.85
Total fluxes	71

References

Bellasio C, Beerling DJ, Griffiths H. 2016. Deriving C4 photosynthetic parameters from combined gas exchange and chlorophyll fluorescence using an Excel tool: theory and practice. *Plant, Cell & Environment* **39**, 1164–1179.

Bellasio C, Burgess SJ, Griffiths H, Hibberd JM. 2014. A high throughput gas exchange screen for determining rates of photorespiration or regulation of C4 activity. *Journal of Experimental Botany* **65**, 3769-3779.

Bellasio C, Griffiths H. 2014. The operation of two decarboxylases (NADPME and PEPCK), transamination and partitioning of C4 metabolic processes between mesophyll and bundle sheath cells allows light capture to be balanced for the maize C4 pathway. *Plant Physiology* **164**, 466-480.

Buckley TN, Adams MA. 2011. An analytical model of non-photorespiratory CO2 release in the light and dark in leaves of C3 species based on stoichiometric flux balance. *Plant, Cell & Environment* **34**, 89-112.

Koteyeva NK, Voznesenskaya EV, Edwards GE. 2015. An assessment of the capacity for phosphoenolpyruvate carboxykinase to contribute to C4 photosynthesis. *Plant Science* **235**, 70-80.

Leegood RC, Walker AP. 1999. Regulation of the C4 pathway. In: Sage RF, Monson RK, eds. *C4 plant biology*. San Diego: Academic Press.

Mallmann J, Heckmann D, Brautigam A, Lercher MJ, Weber APM, Westhoff P, Gowik U. 2014. The role of photorespiration during the evolution of C4 photosynthesis in the genus Flaveria. *Elife* **3**, 10.7554/eLife.02478.

Pick TR, Brautigam A, Schluter U, Denton AK, Colmsee C, Scholz U, Fahnenstich H, Pieruschka R, Rascher U, Sonnewald U, Weber APM. 2011. Systems Analysis of a Maize Leaf Developmental Gradient Redefines the Current C-4 Model and Provides Candidates for Regulation. *Plant Cell* **23**, 4208-4220.

Smith AM, Woolhouse HW. 1983. Metabolism of Phosphoenolpyruvate in the C4 Cycle during Photosynthesis in the Phosphoenolpyruvate-Carboxykinase C4 Grass Spartina-Anglica Hubb. *Planta* **159**, 570-578.

Stutz SS, Edwards GE, Cousins AB. 2014. Single-cell C4 photosynthesis: efficiency and acclimation of Bienertia sinuspersici to growth under low light. *New Phytologist* **202**, 220-232.

von Caemmerer S. 2000. *Biochemical models of leaf Photosynthesis*. Collingwood: CSIRO Publishing.

Wang Y, Long SP, Zhu X-G. 2014. Elements Required for an Efficient NADP-Malic Enzyme Type C4 Photosynthesis. *Plant Physiology* **164**, 2231-2246.
